# Preterm Birth and Cardiometabolic Health Trajectories From Birth to Adulthood: The Avon Longitudinal Study of Parents and Children

**DOI:** 10.1161/JAHA.123.030823

**Published:** 2025-02-03

**Authors:** Gemma L. Clayton, Laura D. Howe, Linda M. O'Keeffe, Adam J. Lewandowski, Deborah A. Lawlor, Abigail Fraser

**Affiliations:** ^1^ Medical Research Council Integrative Epidemiology Unit at the University of Bristol Bristol UK; ^2^ Population Health Science, Bristol Medical School University of Bristol Bristol UK; ^3^ School of Public Health University College Cork Cork Ireland; ^4^ Nuffield Department of Population Health University of Oxford Oxford UK; ^5^ Bristol The National Institute for Health Research Biomedical Research Centre Bristol UK

**Keywords:** ALSPAC, birth weight, cardiometabolic, cardiovascular, preterm, Cardiovascular Disease, Epidemiology

## Abstract

**Background:**

Adults who were born prematurely (<37 weeks’ gestation) are at increased cardiovascular disease risk, but it is unclear when in the life course this risk emerges. Our aim was to compare trajectories of multiple cardiometabolic risk factors from childhood to early adulthood between those who had and had not been born preterm.

**Methods and Results:**

Multilevel models were used to compare trajectories from early childhood (<9 years) to age 25 years of body mass index, fat and lean mass, systolic and diastolic blood pressure, lipids, glucose, and insulin, between individuals born preterm (N=311–676; range, 25–36 weeks’ gestation) and term (N=4973–10 534) in a UK birth cohort study. We also investigated gestational age as a continuum. In children born preterm (versus term), systolic and diastolic blood pressures were higher at age 7 years (mean predicted differences, 0.7 [95% CI, −0.2 to 1.6] mm Hg and 0.6 [95% CI, −0.04 to 1.3] mm Hg, respectively). By age 18 years, the difference in systolic blood pressure persisted (1.9 [95% CI, 0.8–3.1] mm Hg) and in diastolic blood pressure (0.1 [95% CI, −0.7 to 1.0 mm Hg]) disappeared. By age 25 years, this difference in systolic blood pressure began to attenuate towards the null (0.9 [95% CI, −0.5 to 2.3] mm Hg). Participants born preterm (versus term) had lower body mass index between ages 7 and 18 years, but by age 25 years, there was no difference. Fat and lean mass trajectories were consistent with body mass index. High‐density lipoprotein cholesterol was higher and triglycerides lower at birth, in those born preterm, but this difference also disappeared by age 25 years. There was no evidence of differences in glucose and insulin.

**Conclusions:**

Few, modest differences in cardiometabolic health were found in those born preterm versus term. All disappeared by age 25 years, except the small difference in systolic blood pressure. Longer follow‐up is needed to establish if and when cardiometabolic health trajectories diverge between these 2 groups.

**Nonstandard Abbreviations and Acronyms**
ALSPAC: Avon Longitudinal Study of Parents and Children
DBP: diastolic blood pressure
HDP: hypertensive disorders of pregnancy
SBP: systolic blood pressure

**Clinical Perspective**

**What Is New?**

- Whether life course trajectories of commonly assessed cardiovascular disease risk factors, such as blood pressure, lipids, etc, are different in people born preterm versus those born at term, is unknown.
- By age 25 years, we generally found no evidence of differences between people born preterm and term on measures of cardiometabolic health, except that systolic blood pressure was modestly higher in those born preterm; we also observed more favorable outcomes with lower adiposity measures between ages 9 and 18 years.

**What Are the Clinical Implications?**

- The reported increased risk of cardiovascular disease in people born preterm is not apparent in early adulthood.
- Although it may emerge in later life, our results suggest no justification for cardiovascular disease screening using “classic” risk factors in young adults based on their gestational age.
- Further work to replicate these findings in other independent cohorts and studies with follow‐up into midlife are required to examine when associations emerge.

Some 10% of babies worldwide are born preterm (defined as <37 completed weeks of gestation).^1^ Several national registry linkage studies have found that adults born preterm are at increased risk of cardiovascular disease (CVDs), including stroke and myocardial infarction.^2^, ^3^, ^4^, ^5^ Associations between preterm birth and adverse levels of CVD risk factors in early adulthood, such as an increased risk of hypertension,^6^, ^7^ higher diastolic blood pressure,^8^, ^9^ higher lipid levels,^4^, ^10^, ^11^ and higher body mass index (BMI),^12^, ^13^, ^14^, ^15^ have also been reported. A recent systematic review reported that adverse cardiometabolic consequences may not be limited to extreme and very preterm individuals (born before 28 and between 28 and 32 weeks, respectively), with some evidence of moderate to late preterm birth (between 32 and 36 weeks gestation) also associated with an increased risk of hypertension and diabetes.^16^ Studies have also reported that individuals born preterm have a more adverse cardiac structure and function compared with those born at full term.^17^, ^18^ However, studies with these more detailed measurements also tend to have limited sample sizes.

Risk of preterm birth is associated with multiple maternal and demographic factors,^5^, ^7^, ^19^ many of which are also potential confounders in studies of preterm birth and future CVD. This can include older maternal age, smoking during pregnancy, low socioeconomic status, and certain medical conditions, such as, hypertension and diabetes. Although there is consistent evidence linking preterm birth with CVD and its risk factors later in life, it is less clear when these associations emerge in the life course. Therefore, it is important to explore how being born preterm and the development of risk factors in the early stages of life can contribute to understanding the mechanisms and pathways that cause an increased risk of CVD in later life. Specifically, we need detailed repeated measures to identify which risk factors are involved in this process and when these associations begin to emerge. However, when looking at risk factors in earlier life, the focus is on early onset of CVD using cardiometabolic risk factors rather than occurrences of CVD, such as myocardial infarction and stroke, that are more likely to occur later in life. As many cardiometabolic risk factors in early life are modifiable, understanding if and when these associations emerge can facilitate the early detection of potential CVD and enable proactive intervention.

The aim of this study is to compare trajectories of multiple cardiometabolic risk factors from childhood to early adulthood between individuals born preterm and those born full‐term in the early 1990s. We also examine gestational age as a continuum. The cardiometabolic risk factors that we study are BMI, fat and lean mass, systolic blood pressure (SBP), diastolic blood pressure (DBP), pulse rate, triglycerides, high‐density lipoprotein cholesterol (HDL‐C), non–HDL‐C, glucose, and insulin.

## METHODS

### Availability of Data and Materials

Consent for biological samples has been collected in accordance with the Human Tissue Act (2004). Informed consent for the use of data collected via questionnaires and clinics was obtained from participants following the recommendations of the ALSPAC (Avon Longitudinal Study of Parents and Children) Ethics and Law Committee at the time (http://www.bristol.ac.uk/alspac/researchers/research‐ethics/).

Access to ALSPAC data is through a system of managed open access (http://www.bristol.ac.uk/alspac/researchers/access/).

Analysis scripts can be found on the following GitHub page: https://github.com/gc13313/CVDinpretermoffspring.

### Study Participants

Data from the ALSPAC study were used. All pregnant women resident in the area surrounding the city of Bristol, United Kingdom, who had an estimated delivery date between April 1, 1991, and December 31, 1992, were eligible for the study.^20^ Briefly, ALSPAC initially enrolled a cohort of 14 541 pregnancies, from which 14 062 live births occurred, and 13 988 children were alive at 1 year of age. When the oldest children were ≈7 years of age, an attempt was made to bolster the initial sample with eligible cases who had failed to join the study originally. The total sample size for analyses using any data collected after the age of 7 years is therefore 15 447 pregnancies. Of these, 14 901 children were alive at 1 year of age. Follow‐up has included parent and child completed questionnaires, links to routine data, and clinic attendance. Research clinics were held when the participants were ≈7, 9, 10, 11, 13, 15, 18, and 25 years old. Study data were collected and managed using Research Electronic Data Capture electronic data capture tools hosted at the University of Bristol. Research Electronic Data Capture is a secure, web‐based software platform designed to support data capture for research studies.^21^ Full details of recruitment, follow‐up, and data collection for these women have been reported elsewhere.^20^, ^22^, ^23^ Please note that the study website contains details of all the data that are available through a fully searchable data dictionary and variable search tool: http://www.bristol.ac.uk/alspac/researchers/our‐data/. Ethical approval for the study was obtained from the ALSPAC Ethics and Law Committee and the Local Research Ethics Committees. We included singleton pregnancies that resulted in a livebirth and had at least 1 of our cardiometabolic risk factors of interest (described below). We also restricted our main analysis to <42 weeks and excluded (offspring) female participants from the 18‐ and 25‐year clinics who were pregnant at the time, resulting in N=11 896.

### Gestational Age

Gestational age was determined from the last menstrual period but adjusted to reflect the early pregnancy ultrasound estimation if the 2 differed by ≥2 weeks, according to the clinical protocol in use at the time. Preterm birth was defined as delivery ≥24 and <37 completed weeks of gestation. Pregnancies with a recorded gestational age of >41 weeks were excluded (and included in a sensitivity analysis) as there is evidence of worse fetal outcomes in prolonged gestations.^24^

### Study Outcomes

Table 1 summarizes the ages at which each risk factors was assessed and the sample size at each time point.

**Table 1:** Number of Participants With Cardiometabolic Measures at Each Time Point From Birth to 25 Years

Variable	Birth	Age 7 y	Age 9 y	Age 10 y	Age 11 y	Age 12 y	Age 13 y	Age 15 y	Age 18 y	Age 25 y	Total measures	Median (IQR) measures
Age, y	0.0	7.5	9.9	10.6	11.7	12.8	13.8	15.5	17.8	24.5		
Fat/lean mass			6938		6632		5724	4898	4552	3580	32 324	4 (3–6)
SBP/DBP/pulse rate		7618	7197	7086	6678	6398	5097	5056	4385	3703	53 218	7 (4–9)
Glucose		4798	881					3314	3092	3013	10 300	2 (1–3)
Insulin	639	891	4794					3310	3041	3013	15 688	2 (1–3)
Lipids	4707	5108	4823					3314	3092	3013	24 057	2 (1–4)

Lean/fat mass based on information from 6 clinics at ages 9, 11, 13, 15, 18, and 25 y. SBP/DBP/pulse rate based on information from 9 clinics at ages 7, 9, 10, 11, 12, 13, 15, 18, and 25 y. Glucose based on information from 5 clinics at ages 7, 9, 15, 18, and 25 y. Insulin based on information from 6 clinics at ages birth, 7, 9, 15, 18, and 25 y. Lipids based on information from 6 clinics at ages birth, 7, 9, 15, 18, and 25 y. DBP indicates diastolic blood pressure; IQR, interquartile range; and SBP, systolic blood pressure.

### Anthropometry

BMI (weight [kg] divided by height squared [m^2^]) was calculated from 1 to 25 years using data from several sources, including research clinics, routine child health clinics, health visitor records, and questionnaires. Central fat and lean mass were derived from whole body dual‐energy X‐ray absorptiometry scans using a Lunar prodigy narrow fan beam densitometer from 9 to 25 years (at ages 9, 11, 13, 15, 18, and 25 years).

### SBP, DBP, and Pulse Rate

SBP, DBP, and pulse rate were measured from 7 to 25 years (ages 7, 9, 10, 11, 13, 15, 18, and 25 years) at least twice each with the participant sitting and at rest with the arm supported, using a validated device and a cuff size appropriate for the upper arm circumference. The mean of the 2 final measures at each data collection time point was used here.

### Blood‐Based Biomarkers

#### Lipids

Total cholesterol, HDL‐C, and triglycerides were measured in cord blood at birth and from venous blood subsequently at ages 7, 9, 15, 18, and 25 years. Non–HDL‐C was calculated by subtracting HDL‐C from total cholesterol at each measurement occasion. Samples were nonfasted at 7 and 9 years; fasting measures were available from clinics at 15, 18, and 25 years.

#### Insulin and Glucose

Insulin was measured on cord blood at birth. Nonfasting glucose was measured at age 7 years as part of metabolic trait profiling, using nuclear magnetic resonance spectroscopy. Fasting glucose and insulin were also available for a random 10% of the cohort at age 9 years. Fasting glucose and insulin were measured at 15, 18, and 25 years.

### Confounders and Other Variables of Interest

Confounders were defined a priori using directed acyclic diagrams.^25^ Our analyses were adjusted for sex and maternal characteristics, including age, self‐reported prepregnancy BMI (kg/m^2^), smoking (any smoking during pregnancy versus not), parity (0, 1, 2, and ≥3 pregnancies), alcohol intake during pregnancy (any/none), maternal education, hypertensive disorders of pregnancy (HDP) or preexisting hypertension, gestational diabetes, glycosuria, or existing diabetes, ethnicity (White European or non‐White European), and any treatments to help conception in the pregnancy, including in vitro fertilization. Maternal education was defined by the highest attained qualification: (i) certificate of secondary education, ordinary (O) level or vocational certificate (qualifications usually obtained at age 16 years, the UK minimum school leaving age when these women were at school), (ii) advanced A‐level (usually taken at 18 years), or (iii) university degree. Information on prepregnancy BMI, number of previous pregnancies, and maternal education was obtained by self‐report around the time of recruitment (during pregnancy at ≈12 weeks gestation) to the study (mean age, 28.3 years; SD, 4.8 years). Maternal smoking status and alcohol intake before/during pregnancy was also self‐reported in pregnancy questionnaires up to 30 weeks’ gestation. In the United Kingdom and our study population, only women with risk factors (BMI 30 kg/m^2^, south Asian or Middle Eastern ethnicity, family history of diabetes, and previous macrosomia or gestational diabetes) or gestational diabetes at the first antenatal clinic visit would have been invited for a diagnostic oral glucose tolerance test. As the ALSPAC participants were born in the early 1990s when obesity was rare, and the vast majority of our participants were White European, so few (at most 56 cases) were diagnosed with gestational diabetes. We therefore grouped women with evidence of glycosuria (which could be indicative of diabetes) and existing diabetes before pregnancy. HDP was defined from SBP and DBP measurements, along with proteinuria events, for women who consented to obstetric data abstraction. Research midwives collated all measurements, applying the 1988 International Society for the Study of Hypertension in Pregnancy criteria.

We performed additional sensitivity analyses adjusting for birth weight to explore whether we were primarily capturing differences in preterm birth or differences in birth weight. Birth weight data were recorded by ALSPAC research staff after delivery or abstracted from medical records. However, given the intrinsic link between gestational age and birth weight, the classification of birth weight as a confounder or a mediator is debatable.

### Missing Data

Missing values of maternal confounders were imputed because of ~35% (4477/12 830) missingness (Table S1). These were imputed using multivariable multiple imputation with chained equations, performed using the mi impute command in Stata 16. Those with complete data were more likely to have a higher education, more likely to be older, and less likely to smoke or drink alcohol during pregnancy (Table S1). We used 50 imputed data sets (with 25 iterations) and included all variables in the imputation models along with several additional auxiliary variables (to help improve the prediction of missing values) (Table S2). The amount of missing data and the characteristics before and after imputation are presented in Table S3.

### Statistical Analysis

We used multilevel models to examine and compare trajectories of cardiometabolic health of participants born preterm with those born at term, and by gestational age as a continuous variable. Multilevel models estimate mean trajectories of the outcome while accounting for nonindependence of measures within individuals and include all participants with at least 1 cardiometabolic measure, under the missing at random assumption.

Trajectories of cardiometabolic health up to age 18 years by sex were modeled previously.^26^, ^27^ All trajectories except BMI were estimated using linear spline multilevel models, and trajectories of BMI were modeled using fractional polynomial multilevel models. Briefly, linear splines allow knot points to be fit at different ages to derive periods in which change is approximately linear (between knots) whilst fractional polynomials can allow for more complex relationships and involve raising age to many combinations of powers. Individual‐level random effects allow the intercepts and slopes for each period to differ between individuals and therefore each model was fit with random effects at each knot point. For lean mass, because of few available repeated measures, we modeled the person‐specific random effects as a single linear slope rather than a function of the splines, as was done in all other linear spline models. This allowed person‐specific variation from the average trajectory, but under the assumption that person‐specific deviation from the mean trajectory was constant over time. We did not put any constraints on the individual‐level variance covariance matrix (ie, distinct variances and covariances were allowed) as (a priori) it is unlikely that between‐individual variation would remain constant across the observed age range. An age‐ and height‐adjusted covariate was included as a fixed effect in fat/lean mass models.

Here, we extended the existing models^26^ to incorporate data from the 25‐year clinic. To do this, we examined whether an additional knot point was needed at 18 years for all risk factors except BMI (the placement of knots is described in Table S4). Model fit statistics, including Akaike information criterion and Bayesian information criterion, were used to identify the best fitting model. The model with the lowest Akaike information criterion and Bayesian information criterion values was chosen as the best fitting model (Table S5). With the addition of the 25‐year data for BMI, we used fractional polynomials again to help select the best fitting curve. Briefly, BMI was log transformed because of skewness of the data, and fractional polynomials were used where age was raised to various combinations of powers (each of the following single powers, plus each combination of 2 powers: 0.5, 1, 2, 3, −0.5, −1, −2, natural log), from which we selected the best‐fitting curve (the one with the lowest likelihood value). The resulting curve contained 3 age terms, including log age, log age*age, and log age*age^2^.

Models were fit with an interaction term between age and an indicator for preterm (versus full term) or gestational age, adjusting for sex and maternal confounders as detailed earlier. Sex and maternal confounders were added into the model as main effects and interacted with time, which adjusts for the association between the covariate and the outcome at baseline and additionally over time (offspring age). Values of cardiometabolic risk factors that had a skewed distribution (BMI, fat mass, insulin, and triglyceride) were (natural) log transformed before analysis. We report these associations both as an absolute association (on the log scale) and the relative measure as a percentage (difference on the log scale, multiplied by 100). Fat mass and lean mass were adjusted for height using the time‐ and sex‐varying power of height that best resulted in a height‐invariant measure to ensure at all ages lean and fat mass are independent of height.^26^, ^28^ All trajectories were modeled in MLwiN version 3.04, called from Stata version 16 using the runmlwin command. Model fit was assessed by comparing the mean observed values to the mean predicted values for each outcome by preterm/full term across age. We report the adjusted estimates for maternal age, smoking, alcohol, BMI, parity, sex, education, HDP, gestational diabetes, ethnicity, and any intervention to help conception from the multiple imputation data sets as the main results.

To ascertain whether or not the associations between gestational age (continuous) and each outcome were linear within each spline period, we compared models using a likelihood ratio test with gestational age split into quartiles and included as a single variable with models in which quartiles of gestational age was included as 3 dummy variables. These terms were fit as interactions with age within each spline period.

As suggested by a reviewer, given that there are sex differences in many of the cardiometabolic outcomes from birth to early adulthood, we also present the main analysis stratified by sex. We conducted several sensitivity analyses. We report both unadjusted and adjusted results following multiple imputation (adjusted are presented as the main results). We also report a complete case analysis. We included births >41 gestational weeks (n up to 934) to evaluate potential selection bias arising from excluding these participants from our main analysis. We could not distinguish between medically indicated and spontaneous preterm births, but in a sensitivity analysis, we restricted to spontaneous preterm births, which are likely to exclude most medically indicated preterm births. We also ran a model in which we additionally adjusted for birth weight to investigate whether any associations between gestational age and outcomes were driven by size at birth.

## RESULTS

Maternal characteristics, categorized by preterm birth (range, 25–36 weeks’ gestation) and full term (range, 37–41 weeks’ gestation), are detailed in Table 2. Individuals born preterm were of a lower birth weight than individuals born at term. Mothers of preterm and term babies were similar ages (27.8 versus 28.1 years); but a higher proportion of mothers to preterm babies were first‐time mothers and had lower levels of education compared with mothers of term babies. HDP was more common among mother who delivered preterm (24.6% versus 15.6%).

**Table 2:** Summary of Observed Characteristics Between Those Born Preterm and Full Terms

Variable	Preterm birth (N=733)		Full‐term birth* (N=11 163)	
Gestational age, median (IQR), wk	35.0	(34.0–36.0)	40.0	(39.0–40.0)
Maternal age, mean±SD, y	27.8±	4.9	28.2±	4.9
BMI at first assessment				
Normal	476/596	79.9%	7564/9491	79.7%
Overweight	85/596	14.3%	1410/9491	14.9%
Obese	35/596	5.9%	517/9491	5.4%
Any maternal smoking (before or during pregnancy)				
No	514/692	74.3%	8022/10606	75.6%
Yes	178/692	25.7%	2584/10606	24.4%
Parity				
0	342/687	49.8%	4606/10467	44.0%
1	211/687	30.7%	3763/10467	36.0%
2	103/687	15.0%	1495/10467	14.3%
≥3	31/687	4.5%	603/10467	5.8%
Educational achievement				
CSE/vocational/O level	427/639	66.8%	6487/10205	63.6%
A level	143/639	22.4%	2351/10205	23.0%
Degree	69/639	10.8%	1367/10205	13.4%
Ethnicity				
White	691/733	94.3%	10 684/11163	95.7%
Non‐White†	42/733	5.7%	479/11163	4.3%
Diabetes status in pregnancy				
No glycosuria or diabetes	602/644	93.5%	9745/10152	96.0%
Existing diabetes	14/644	2.2%	30/10152	0.3%
Gestational diabetes	10/644	1.6%	45/10152	0.4%
Glycosuria	18/644	2.8%	332/10152	3.3%
HDP	180/731	24.6%	1728/11075	15.6%
Previous hypertension	32/631	5.1%	366/9942	3.7%
Any pregnancy complications (HDP or preexisting hypertension or GDM or glycosuria)	232/631	36.8%	2386/9942	24.0%
Any treatments to help conception in this pregnancy (including IVF)‡	39/733	5.3%	309/11163	2.8%
Pregnancy at 18‐y clinic	1/733	0.1%	10/11163	0.1%
Pregnancy at 25‐y clinic	1/733	0.1%	16/11163	0.1%
Low birth weight (<2500 g)	385/718	53.6%	255/11034	2.3%

Numbers are based on those included in the sample with at least 1 risk factor (outcome). The number of preterms and full‐term births are summarized by outcome and analysis in Table S26. BMI indicates body mass index; CSE, certificate of secondary education; GDM, gestational diabetes; HDP, hypertensive disorders of pregnancy; IQR, interquartile range; and IVF, in vitro fertilization.

* Restricted to the main analysis population of <42 weeks’ gestation.

^†^ Non‐White includes: Black Caribbean; Black African; Black Other; Indian; Pakistani; Bangladeshi; Chinese; Any Other.

^‡^ This included those who had intrauterine insemination, ovulation induction, and male infertility treatment/other infertility treatment. At the 25‐year clinic, 14 individuals reported diabetes, of which, all 14 were born full term. A total of 18 individuals reported “heart disease or heart problems,” of which, all 15 were born full term and 3 born preterm. Of these 32, 26 were taking medication, of which 2 of 26 were taking β‐blockers (although it is unknown if prescribed for hypertension or something else). Because of these small number and because they cannot be confounders, medication use was not accounted for in analyses.

**Figure 1:**
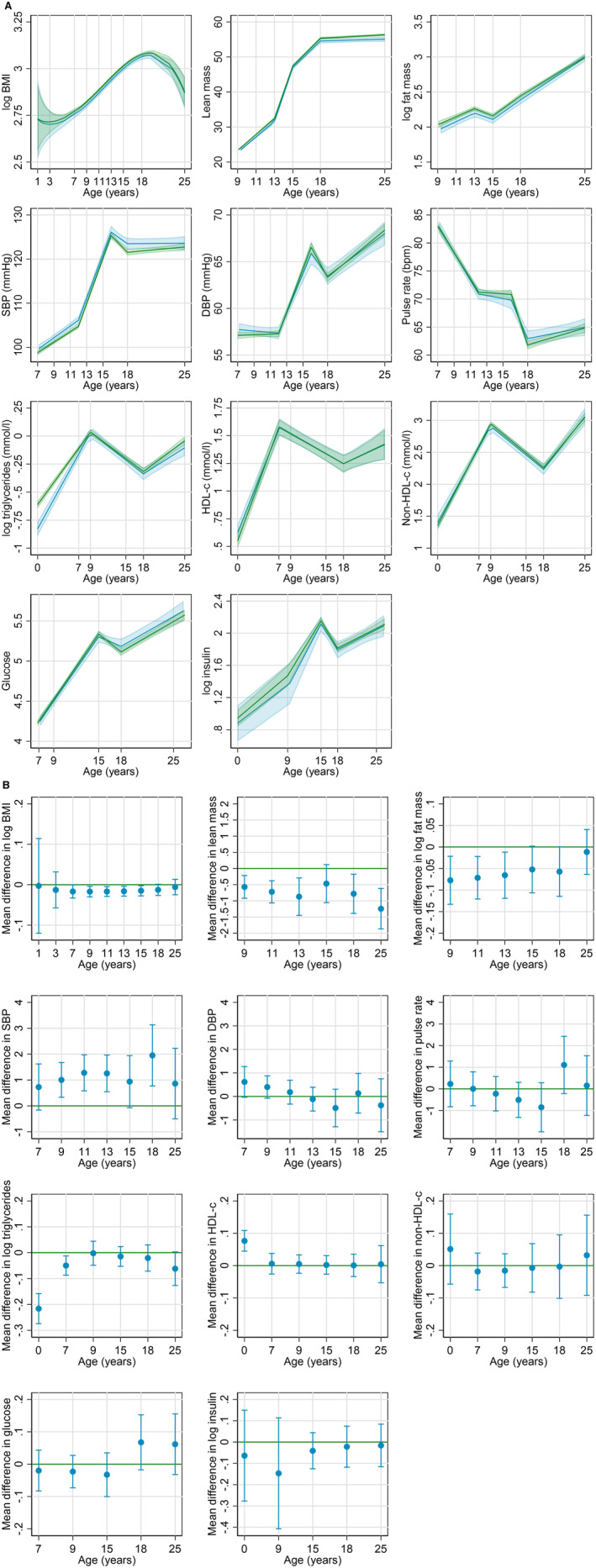
Predicted mean (95% CI) trajectories of cardiometabolic risk factors by preterm (blue) and full‐term (green) births (A) and estimated mean difference (95% CI) between those born preterm versus full term (B). Full terms restricted to <42 wks. Preterm/full‐term numbers for body mass index (BMI), lean/fat mass, systolic blood pressure (SBP)/diastolic blood pressure (DBP)/pulse rate, triglycerides/high‐density lipoprotein (HDL)/low‐density lipoprotein, glucose, and insulin were: 676/10 534, 493/7380, 559/8195, 542/8566, 417/6339, and 311/4973. All models adjusted for (and entered into the models as main effects and interactions with offspring age) offspring sex and maternal characteristics: age, parity, education, smoking status, alcohol intake, prepregnancy BMI, hypertensive disorders of pregnancy (HDP) or preexisting hypertension, existing diabetes, gestational diabetes (GDM), or glycosuria, ethnicity (White European compared with non‐White European), and any treatments to help conception in this pregnancy (including in vitro fertilization) (compared with none). Trajectories drawn for female participants, White European ethnicity, mean maternal age, and prepregnancy BMI, no HDP or preexisting hypertension, no previous children, no maternal smoking or alcohol, educated to certificate of secondary education/vocational/O level, no existing diabetes, GDM, or glycosuria, and no treatments to help conceive. Estimates <0 mean lower values for preterm versus term. (**B**) The mean difference (with 95% CI) in individuals born preterm (compared with full term). HDL‐C indicates HDL cholesterol.

**Figure 2:**
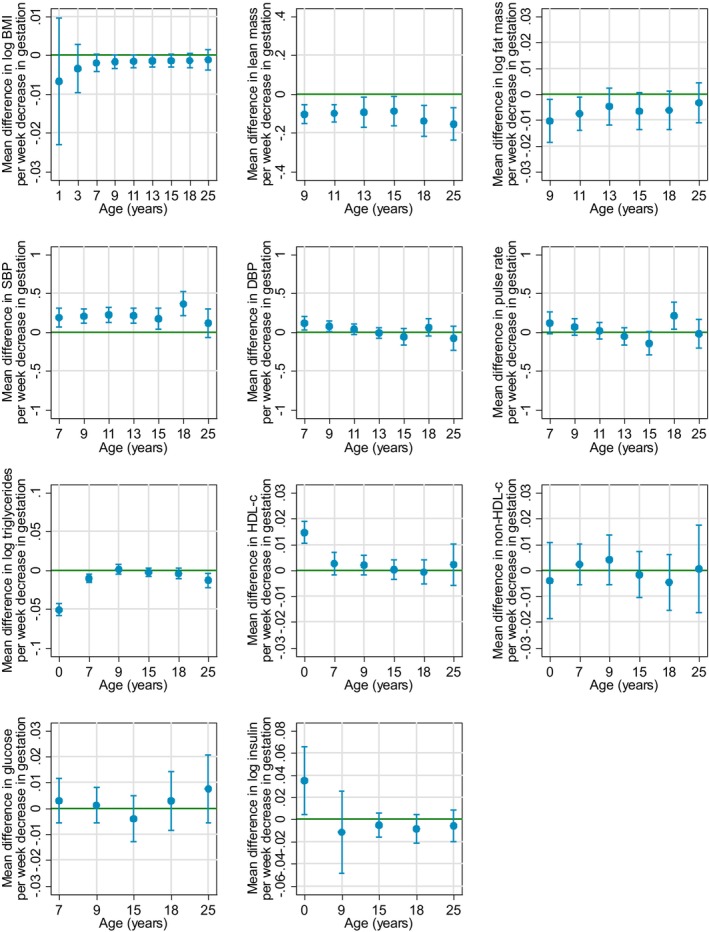
Estimated mean difference in cardiometabolic risk factors per 1‐week decrease in gestational age. Gestational age restricted to <42 wks. All models adjusted for (and entered into the models as main effects and interactions with offspring age) offspring sex and maternal characteristics: age, parity, education, smoking status, alcohol intake, prepregnancy body mass index (BMI), hypertensive disorders of pregnancy or preexisting hypertension, existing diabetes, gestational diabetes, or glycosuria, ethnicity (White European compared with non‐White European), and any treatments to help conception in this pregnancy (including in vitro fertilization) (compared with none). This figure shows the mean difference (with 95% CI) per 1‐wk decrease in gestational age. DBP indicates diastolic blood pressure; HDL‐C, high‐density lipoprotein cholesterol; and SBP, systolic blood pressure.

There was no evidence for a departure from linearity in the association between gestational age (continuous) and all outcomes (Table S6). Figures 1 and 2 show associations of preterm birth and gestational age with cardiovascular risk factors, respectively, adjusted for all measured confounders (coefficients are presented in Tables S7–S10). Figure S1 presents the associations of preterm birth adjusted for birth weight. Results from other models (sensitivity analyses) are presented in Tables S11 to S27. Observed and predicted values across each spline period and first and last time points were similar for all outcomes (Tables S22–S25).

Overall, the trajectory of BMI from birth to early adulthood was J shaped, decreasing from age 1 to 7 years, followed by an increase until age 18 years, and subsequently plateauing by age 25 years (Figure 1A and Table S11). The predicted mean BMI was similar in participants born preterm compared with those born at term at ages 1, 3, and 25 years, whereas participants born preterm had a lower mean BMI between the ages of 7 and 18 years (Figure 1B). (Height‐adjusted) lean and fat mass (measured from age 9 years only) trajectories were consistent with BMI. For example, mean fat mass was 5% (95% confidence interval (CI), (0%–11%)) lower in preterm participants compared with those born at term, at age 15 years. Results were consistent when gestational age was modeled continuously (Figure 2, Table S26) and when analyses included pregnancies of 42 weeks or more (Tables S11–S13).

SBP and DBP generally increased from age 7 to 16 years, followed by a decrease until age 18 years and then beginning to plateau (Figure 1A). Both SBP and DBP were higher at age 7 years (mean predicted differences, 0.7 [95% CI, −0.2 to 1.6] mm Hg and 0.6 [95% CI, −0.01 to 1.3] mm Hg, respectively) in preterm participants compared with term participants (Figure 1B). From age 16 to 18 years, SBP decreased in both groups but at a slower rate in preterm participants (Table S8). By age 18 years, mean SBP was 1.9 mm Hg higher (95% CI, 0.8–3.1 mm Hg) in preterm participants (Table S14). The difference in SBP persisted at age 25 years (0.9 [95% CI, −0.5 to 2.3] mm Hg) but the lower bound of the CI crossed the null, whereas the difference in DBP attenuated to the null (−0.3 [95% CI, −1.5 to 0.8] mm Hg). When modeling gestational age as a continuum (Figure 2, Table S26), these differences were also apparent; for example, mean SBP was 0.36 mm Hg (95% CI, 0.21–0.52 mm Hg) and 0.11 mm Hg (95% CI, −0.07 to 0.30 mm Hg) higher at age 18 and age 25 years, respectively, per 1‐week decrease in gestational age.

HDL‐C was higher, and triglycerides lower, at birth in those born preterm, but this difference persisted for triglycerides and attenuated to the null for HDL‐C by 25 years (Figure 1B). Glucose appeared to be slightly higher at ages 18 and 25 years (Figure 1B). No other differences were found (Figures 1 and 2). Furthermore, results were consistent when gestational was modeled continuously (Figure 2, Table S26).

In sensitivity analyses, when pregnancies >41 weeks were included (Tables S11–S21), results were consistent. Results were also similar in the complete case and multiple imputation analyses (Tables S11–S21). When adjusting for birth weight, results remained similar across all risk factors (Figure S1). For example, results attenuated at later ages for glucose, suggesting birth weight could be an explanation whilst they remained for most ages of SBP, suggesting preterm birth is likely driving this association. Overall, we see that the association of preterm versus full‐term birth is similar for female participants and male participants for most cardiometabolic risk factors with overlapping CIs (Figure S2 contrasts the difference in cardiometabolic trajectories between preterm and term in female participants and male participants separately). When we restricted the analysis to include only spontaneous preterm births (~42% of preterm births were excluded across outcomes), results were consistent with the main analysis (Figure S3).

## DISCUSSION

### Main Findings

In this article, we examined longitudinal changes in 11 measures of cardiometabolic health from early childhood or birth through to 25 years in participants born preterm compared with those born at term in a large contemporary birth cohort study. Some differences during early life were noted (namely, lower height‐adjusted lean and fat mass at age 9 years and marginally higher SBP throughout from age 7 to 25 years). Most of these differences attenuated to the null by age 25 years, but a small difference in mean SBP (~1 mm Hg higher) was observed in participants born preterm compared with term.

Given that we know that preterm born individuals have a higher risk of CVD,^3^, ^7^ several explanations for our results are possible. It is possible that in early life the cardiovascular risk factors measured here do not capture an already increased cardiovascular risk in people born preterm, and are better reflected using imaging modalities, such as retinopathy, which have been shown to differ in adolescents by gestational age.^9^ These differences may be reflected in divergent trajectories of blood pressure and other cardiometabolic risk factors measured here after 25 years and in later adulthood (up to 37 years), and further follow‐up of this and similar cohorts will therefore be valuable.

There are multiple causes of preterm birth (eg, spontaneous preterm birth may involve genetic factors or result from an infection, whereas medically indicated preterm birth may result from maternal complications, such as preeclampsia, that may also impact the cardiovascular health of the offspring).^19^, ^29^ Although we were unable to distinguish between spontaneous and medically indicated preterm birth, in a sensitivity analysis, we restricted the analysis to include only spontaneous preterm births, which is likely to exclude most medically indicated preterm births related to pregnancy complications, and saw consistent results with overall preterm birth. Multiple maternal health‐related behaviors and conditions before and during pregnancy, such as obesity and smoking, are known to be associated with the risk of preterm birth^30^ and may also affect offspring cardiometabolic traits either via inherited genetic variation or attributable to shared familial environmental exposures.^31^, ^32^ Studies have shown that genetic variants associated with maternal blood pressure are also associated with a shorter duration of gestation and preterm birth.^32^ This could mean that the association we find here between preterm birth and higher SBP is driven by genetic confounding (with offspring inheriting maternal genetic variants linked to higher blood pressure) rather than by exposure to preterm birth itself. Recent evidence from sibling and Mendelian randomization studies^33^, ^34^ shows that it is likely not the direct in utero exposure with HDP that affects the offspring cardiometabolic health as levels were similar in siblings exposed and unexposed to HDP in utero.

We also found evidence of favorable (lower) adiposity measures between ages 9 and 18 years in people born preterm. Although initially counterintuitive to higher blood pressure, this inverse relationship of lower birth weight and higher later blood pressure has been attributed to genetic effects.^35^

### Comparison to Other Studies

To the best of our knowledge, no other study has examined trajectories of cardiometabolic risk factors over time in adults born preterm versus term or by gestational age. Our findings suggest that those born preterm have a lower lean and fat mass at 9 years compared with full terms and are in line with other studies.^36^, ^37^, ^38^ For example, Fewtrell et al measured body composition using dual‐energy X‐ray absorptiometry in 497 children born preterm (gestational age at birth: 25–36 weeks), compared them with 95 term‐born controls at the age of 8.8 to 12.7 years (mean age, 11.2 years) and showed fat mass was lower in preterms.

Our findings are also broadly consistent (although we see a much weaker association) with a population‐based cohort study of individuals born in 1985 to 1989 in northern Finland,^39^ which found that preterm birth is associated with higher SBP at mean age 23 (SD, 1.4) years in a study of 134 preterm participants (3.2 mm Hg [95% CI, 1.1–5.4 mm Hg]) and 242 late preterms (1.5 mm Hg [95% CI, −0.3 to 3.3 mm Hg]) compared with 334 term born participants. A meta‐analysis by Parkinson et al, which included 17 030 preterm and 295 261 term‐born adults from 27 studies, also found higher blood pressure in those born preterm compared with full term.^37^ Bertagnolli et al suggest that the higher SBP in those born preterm (compared with full term) arises from greater vascular stiffness in adults born preterm.^12^ Elastin synthesis in arterial walls occurs at the end of gestation, and a shorter gestation can disrupt this process and cause elastin disruption, which in turn increases vascular stiffness, leading to an increase in SBP and ultimately greater risk of hypertension. The meta‐analysis by Parkinson et al also showed no evidence of an association in insulin or glucose between those born preterm compared with full term.^37^

### Strengths and Limitations

Strengths of this study include the availability of repeat measures of multiple cardiometabolic risk factors over a 25‐year period in a large contemporary, population‐based cohort, and the ability to look at change over time. We also used multiple imputation to account for missing confounder data and included participants with at least 1 measurement in the analysis under the missing at random assumption, so that selection bias was minimized. In our main analysis, we compared participants born preterm (<37 weeks) to term (37–41 weeks). However, in sensitivity analyses, we excluded up to 934 women with a gestational age >41 weeks and our results remained similar. We also note that participants in ALSPAC are predominantly of White European origin, and previous studies have shown ethnic differences in both preterm birth rates and offspring cardiometabolic risk factors, so our findings might not be generalizable to other race and ethnic groups. Nevertheless, our findings are similar to those observed in middle‐income settings in a Brazilian population of similar age.^40^

Despite the size of the cohort, we were unable to stratify preterm birth further into very/extremely preterm because of the small number of participants (N=6 to N=115 across outcomes of those <28 weeks’ gestation). However, we did examine gestational age as a continuous variable. We checked for a departure from a linear association between gestational age in weeks and the cardiometabolic health outcomes and did not find strong evidence of this. Our results across all outcomes were still apparent and estimated more precisely compared with when gestational age was categorized as preterm/full term. However, we acknowledge this may not help if extreme preterms are different and given the small numbers, it is unlikely we would find statistical evidence of departure from linearity even if it is real. Given that previous studies have shown sex differences in cardiometabolic risk factors, we also presented associations of preterm birth with cardiometabolic trajectories by sex. We generally observed that the association of preterm versus full‐term birth was similar for female participants and male participants for most cardiometabolic risk factors with CIs overlapping. Given the relatively small number of people born preterm (140–330 in female participants and 171–403 in male participants) in our cohort, it is likely we are underpowered to fully explore sex differences and larger studies might find some evidence of differences in the trajectories we have explored.

Given that low birth weight is most often caused by being born preterm, it is possible that rather than observing associations of preterm birth, we are also observing associations between low birth weight and cardiovascular health. It is difficult to unpick gestational age from gestational size because of them being so closely related and that it is likely that cause and effect differ among those born preterm.^41^, ^42^ However, including birth weight in the main model made little to no difference to the gestational age/preterm associations. However, our sample size ranged from N=24 to N=70 across outcomes for low birth weight (<1500 g), indicating that we might have been underpowered to adequately assess the influence of birth weight on our associations with preterm birth.

## Conclusions

The known increased risk of CVD seen in adults born preterm was not apparent based on classic CVD risk factors in our early adulthood cohort, except for a modest increase in systolic blood pressure. We also observed more favorable outcomes with lower adiposity measures between ages 9 and 18 years. Reducing preterm birth would be unlikely to have substantial impact on improving conventional cardiometabolic risk factors during the first 25 years of life. Further work to replicate these findings in other independent cohorts and studies with follow‐up into midlife is required to examine when associations emerge.

## Sources of Funding

The UK Medical Research Council and Wellcome (grant 217065/Z/19/Z) and the University of Bristol provide core support for ALSPAC (Avon Longitudinal Study of Parents and Children), with additional support from a wide range of national and international funders (a comprehensive list of grant funding is available on the ALSPAC website; http://www.bristol.ac.uk/alspac/external/documents/grant‐acknowledgements.pdf). This publication is the work of the authors, and all authors will serve as guarantors for the contents of this article. This research was funded in whole, or in part, by the Wellcome Trust (grant 102215/2/13/2). For the purpose of Open Access, the author has applied a CC BY public copyright license to any Author Accepted Manuscript version arising from this submission.

The European Union's Horizon 2020 research and innovation programme under grant agreement 733206 (LifeCycle) funds Dr Clayton's salary. Drs Clayton, Fraser, Howe, and Lawlor work in, or are affiliated with, a unit that is funded by the UK Medical Research Council (grant MC_UU_00011/6) and University of Bristol, and Dr Lawlor is a National Institute for Health Research Senior Investigator (NF‐0616‐10 102) and BHF Chair (CH/F/20/90003). Dr Fraser is funded by a UK MRC fellowship (MR/M009351/1). The funders had no role in study design, data collection and analysis, decision to publish, or preparation of the manuscript. Dr Lewandowski was funded by a BHF Intermediate Research Fellowship (FS/18/3/33292). Dr O'Keeffe is supported by a Health Research Board of Ireland Emerging Investigator Award (grant EIA‐FA‐2019‐007 SCaRLeT). Dr Howe was supported by Health Foundation grant: Social and economic consequences of health status—Causal inference methods and longitudinal, intergenerational data. This was awarded under the Social and Economic Value of Health programme (award reference 807293) and a Career Development Award from the UK Medical Research Council (MR/M020894/1).

## Disclosures

Professor Lawlor has received support from Roche Diagnostics and Medtronic Ltd in the last 10 years for work unrelated to that presented here. The remaining authors have no disclosures to report.

## Supporting information

Tables S1–S27
Figures S1–S3
