# Supplementary material for: Preterm Birth and Cardiometabolic Health Trajectories From Birth to Adulthood: The Avon Longitudinal Study of Parents and Children
Source: J Am Heart Assoc. 2025 Feb 3;14(3):e030823. doi: 10.1161/JAHA.123.030823 (PMC12074750; doi:10.1161/JAHA.123.030823)

# Supplemental Material

**Table S1. Characteristics of those included/who would be excluded through missing covariate data.**

| | | Would be excluded due to missing data | | Complete cases | |
|---|---|---|---|---|---|
| Gestational age, weeks | (median IQR) | 40.0 | (39.0, 41.0) | 40.0 | (39.0, 41.0) |
| Maternal age at delivery, years | (mean, SD) | 27.4 | 5.1 | 28.5 | 4.8 |
| BMI at first assessment | normal | 2006/2502 | 80.2% | 6617/8353 | 79.2% |
| | overweight | 365/2502 | 14.6% | 1266/8353 | 15.2% |
| | obese | 131/2502 | 5.2% | 470/8353 | 5.6% |
| Smoking before or during pregnancy | No | 2725/3820 | 71.3% | 6454/8353 | 77.3% |
| | Yes | 1095/3820 | 28.7% | 1899/8353 | 22.7% |
| Parity | 0 | 1550/3659 | 42.4% | 3837/8353 | 45.9% |
| | 1 | 1270/3659 | 34.7% | 2958/8353 | 35.4% |
| | 2 | 579/3659 | 15.8% | 1127/8353 | 13.5% |
| | 3+ | 260/3659 | 7.1% | 431/8353 | 5.2% |
| Educational achievement | CSE/ Vocational/ O-level | 2282/3340 | 68.3% | 5201/8353 | 62.3% |
| | A level | 685/3340 | 20.5% | 1983/8353 | 23.7% |
| | Degree | 373/3340 | 11.2% | 1169/8353 | 14.0% |
| HDP | | 745/4377 | 17.0% | 1354/8353 | 16.2% |

**Table S2. Variables used in the imputation model.**

| Main model variables (substantive model of interest) | Imputation model |
|---|---|
| **Outcome** | |
| BMI | Linear regression |
| Lean mass, fat mass | Linear regression |
| SBP, DBP, pulse rate | Linear regression |
| Lipids | Linear regression |
| Glucose, insulin | Linear regression |
| **Covariates** | |
| Offspring sex | Logistic regression |
| Maternal age | Linear regression |
| HDP | Logistic regression |
| Maternal BMI | Linear regression |
| Maternal alcohol use during pregnancy | Logistic regression |
| Maternal smoking during pregnancy | Logistic regression |
| Maternal education | Ordinal regression |
| Parity | Ordinal regression |
| **Auxiliary variables [variables not in the substantive model but are either predictive of missingness or the underlying missing values or both]** | |
| Income | Linear regression |
| Pre pregnancy alcohol intake (b720) | Ordinal regression |
| Number of cigarettes per day at 32 weeks (c482) | Ordinal regression |
| Pre pregnancy weight (dw002) | Linear regression |

Each outcome also acts as an auxiliary variable to the other, for example, when the outcome is BMI in the substantive model all outcomes except BMI act as auxiliary variables.

**Table S3. Characteristics of the data before and after imputation.**

| | | % missing | N | Before imputation | After imputation |
|---|---|---|---|---|---|
| | | | | % | % |
| BMI at first assessment | normal | 15.4 | 10855 | 79.4% | 80.6 |
| | overweight | | | 15.0% | 15.7 |
| | obese | | | 5.5% | 3.7 |
| Smoking status | 0 | 5.1 | 12173 | 75.4% | 80.0 |
| | 1 | | | 24.6% | 20.0 |
| Parity | 0 | 6.4 | 12012 | 44.8% | 49.1 |
| | 1 | | | 35.2% | 33.0 |
| | 2 | | | 14.2% | 14.2 |
| | 3+ | | | 5.8% | 3.7 |
| Educational achievement | CSE/ Vocational/ O-level | 8.9 | 11693 | 64.0% | 55.0 |
| | A level | | | 22.8% | 28.0 |
| | Degree | | | 13.2% | 17.0 |

**Table S4 Details of current knot positions up to age 18 years**

| | Age 7 | Age 9 | Age 10 | Age 11 | Age 12 | Age 13 | Age 15 | Age 18 |
|---|---|---|---|---|---|---|---|---|
| SBP/DBP/pulse rate | | | | | x | | x (16) | |
| Fat/lean mass | | | | | | x | x | |
| Glucose | | | | | | | x | |
| Insulin | | x | | | | | x | |
| Non-HDL /triglycerides | | x | | | | | | |
| HDL | x | | | | | | | |

**Table S5 Details of knots when extending models to include 25 year data**

| | | Original knot placements | | | Additional knot at 18 years | | |
|---|---|---|---|---|---|---|---|
| | N | DF | AIC | BIC | DF | AIC | BIC |
| Lean mass | 32217 | 9 | 636512 | 636588 | 10 | 636217 | 636300 |
| Fat mass | 32217 | 16 | 20527 | 20661 | 22 | 19451 | 19635 |
| DBP | 52930 | 15 | 355326 | 355460 | 21 | 354216 | 354402 |
| Pulse rate | 52930 | 15 | 393568 | 393701 | 21 | 391617 | 391803 |
| SBP | 52930 | 15 | 382225 | 382358 | 21 | 381236 | 381423 |
| Triglycerides | 24047 | 10 | 26505 | 26586 | 15 | 25057 | 25179 |
| HDL-c | 23973 | 10 | 7973 | 8054 | 15 | 3795 | 3916 |
| Non-HDL-c | 23973 | 10 | 48980 | 49061 | 15 | 45487 | 45609 |
| Insulin | 15659 | 15 | 32481 | 32595 | 21 | 31973 | 32134 |
| Glucose | 14367 | 10 | 21181 | 21257 | 15 | 20392 | 20505 |

N=observations (not participants); DF=degrees of freedom; AIC= Akaike information criterion; BIC= Bayesian information criterion.

**Table S6 Details of linearity check when gestational age was modelled continuously**

| Outcome | P-value when comparing across 3 categories (very preterm vs preterm vs full term) | P-value when comparing across quartiles |
|---|---|---|
| BMI | 0.0043 | 0.671 |
| Fat mass | 0.0686 | 0.925 |
| Lean mass | 0.5223 | 0.2986 |
| SBP | 0.8181 | 0.5605 |
| DBP | 0.5384 | 0.3959 |
| Pulse rate | 0.3646 | 0.1539 |
| Triglycerides | <0.0001 | 0.0593 |
| HDL-c | 0.0287 | 0.276 |
| Non-HDL-c | 0.0004 | 0.3147 |
| Glucose | 0.7318 | 0.1147 |
| Insulin | 0.3242 | 0.7874 |

**Table S7 Intercept and slope estimates for lean mass and fat mass**

| | Age (years) | Preterm Mean (95% CI) | Term Mean (95% CI) | Mean difference (95% CI) comparing pre-term to term born participants | P value for difference |
|---|---|---|---|---|---|
| Lean mass | Age 9yr (kg) | 22.76 (22.36, 23.15) | 23.33 (23.12, 23.55) | -0.58 (-0.93, -0.22) | 0.0013 |
| | Change 9-13yr (kg/yr) | 2.21 (2.02, 2.40) | 2.28 (2.17, 2.39) | -0.07 (-0.24, 0.09) | 0.3916 |
| | Change 13-15yr (kg/yr) | 7.71 (7.39, 8.03) | 7.51 (7.34, 7.68) | 0.20 (-0.09, 0.49) | 0.1839 |
| | Change 15-18yr (kg/yr) | 2.52 (2.30, 2.74) | 2.62 (2.50, 2.74) | -0.10 (-0.30, 0.10) | 0.3096 |
| | Change 18-25yr (kg/yr) | 0.07 (-0.03, 0.17) | 0.14 (0.09, 0.19) | -0.07 (-0.15, 0.02) | 0.1219 |
| | Age 25yr (kg) | 55.05 (54.35, 55.76) | 56.30 (55.91, 56.69) | -1.25 (-1.88, -0.63) | 0.0001 |
| Fat mass | Age 9yr (kg) | 1.95 (1.88, 2.03) | 2.03 (1.98, 2.08) | -0.08 (-0.13, -0.02) | 0.0058 |
| | Change 9-13yr (kg /yr) | 0.06 (0.04, 0.08) | 0.06 (0.04, 0.07) | 0.00 (-0.01, 0.01) | 0.6106 |
| | Change 13-15yr (kg/yr) | -0.04 (-0.07, -0.02) | -0.05 (-0.06, -0.04) | 0.01 (-0.02, 0.03) | 0.5397 |
| | Change 15-18yr (kg /yr) | 0.09 (0.07, 0.11) | 0.09 (0.08, 0.10) | -0.00 (-0.02, 0.02) | 0.8589 |
| | Change 18-25yr (kg/yr) | 0.09 (0.08, 0.10) | 0.08 (0.07, 0.08) | 0.01 (-0.00, 0.01) | 0.1255 |
| | Age 25yr (kg) | 2.99 (2.93, 3.05) | 3.00 (2.97, 3.03) | -0.01 (-0.06, 0.04) | 0.6569 |

**Table S8 Intercept and slope estimates for SBP, DBP and pulse rate**

| | Age (years) | Preterm Mean (95% CI) | Term Mean (95% CI) | Mean difference (95% CI) comparing pre-term to term born participants | P value for difference |
|---|---|---|---|---|---|
| SBP | Age 7yr (mmHg) | 99.44 (98.45, 100.43) | 98.73 (98.19, 99.26) | 0.71 (-0.18, 1.60) | 0.1167 |
| | Change 7-12yr (mmHg/yr) | 1.34 (1.10, 1.58) | 1.20 (1.07, 1.33) | 0.14 (-0.08, 0.36) | 0.2116 |
| | Change 12-16yr (mmHg/yr) | 4.98 (4.58, 5.38) | 5.14 (4.93, 5.36) | -0.16 (-0.52, 0.20) | 0.3841 |

| | | | | | |
|---|---|---|---|---|---|
| | Change 16-18yr (mmHg/yr) | -1.29 (-2.21, -0.38) | -1.88 (-2.37, -1.40) | 0.59 (-0.24, 1.42) | 0.1620 |
| | Change 18-25yr (mmHg/yr) | 0.03 (-0.22, 0.28) | 0.18 (0.04, 0.32) | -0.15 (-0.38, 0.07) | 0.1887 |
| | Age 25yr (mmHg) | 123.67 (122.13, 125.21) | 122.79 (121.94, 123.63) | 0.89 (-0.48, 2.25) | 0.2017 |
| DBP | Age 7yr (mmHg) | 57.72 (56.99, 58.44) | 57.10 (56.71, 57.49) | 0.61 (-0.04, 1.26) | 0.0658 |
| | Change 7-12yr (mmHg/yr) | -0.07 (-0.26, 0.12) | 0.04 (-0.06, 0.14) | -0.11 (-0.28, 0.06) | 0.2150 |
| | Change 12-16yr (mmHg/yr) | 2.12 (1.77, 2.46) | 2.31 (2.12, 2.49) | -0.19 (-0.50, 0.12) | 0.2335 |
| | Change 16-18yr (mmHg/yr) | -1.19 (-1.95, -0.43) | -1.60 (-2.00, -1.19) | 0.41 (-0.28, 1.10) | 0.2435 |
| | Change 18-25yr (mmHg/yr) | 0.66 (0.46, 0.87) | 0.73 (0.62, 0.84) | -0.07 (-0.25, 0.11) | 0.4602 |
| | Age 25yr (mmHg) | 68.09 (66.82, 69.37) | 68.44 (67.74, 69.13) | -0.34 (-1.47, 0.78) | 0.5488 |
| Pulse rate | Age 7yr (bpm) | 83.20 (82.03, 84.38) | 82.99 (82.34, 83.63) | 0.22 (-0.84, 1.28) | 0.6853 |
| | Change 7-12yr (bpm/yr) | -2.46 (-2.74, -2.18) | -2.35 (-2.50, -2.20) | -0.11 (-0.37, 0.14) | 0.3863 |
| | Change 12-16yr (bpm/yr) | -0.27 (-0.72, 0.17) | -0.10 (-0.34, 0.13) | -0.17 (-0.57, 0.23) | 0.4078 |
| | Change 16-18yr (bpm/yr) | -3.43 (-4.43, -2.42) | -4.49 (-5.03, -3.96) | 1.07 (0.16, 1.98) | 0.0216 |
| | Change 18-25yr (bpm/yr) | 0.28 (0.00, 0.56) | 0.42 (0.27, 0.57) | -0.14 (-0.39, 0.11) | 0.2629 |
| | Age 25yr (bpm) | 64.90 (63.34, 66.47) | 64.78 (63.91, 65.64) | 0.13 (-1.25, 1.51) | 0.8571 |

**Table S9 Intercept and slope estimates for lipids**

| | Age (years) | Preterm Mean (95% CI) | Term Mean (95% CI) | Mean difference (95% CI) comparing pre-term to term born participants | P value for differen ce |
|---|---|---|---|---|---|
| Triglycerides (logged) | Birth (mmol/l) | -0.83 (-0.89, -0.76) | -0.61 (-0.64, -0.58) | -0.22 (-0.27, -0.16) | 0.0000 |
| | Change 0-9yr (mmol/l/yr) | 0.10 (0.09, 0.10) | 0.07 (0.07, 0.08) | 0.02 (0.02, 0.03) | 0.0000 |
| | Change 9-18yr (mmol/l/yr) | -0.04 (-0.05, -0.03) | -0.04 (-0.04, -0.03) | -0.00 (-0.01, 0.01) | 0.5807 |
| | Change 18-25yr (mmol/l/yr) | 0.03 (0.02, 0.04) | 0.04 (0.03, 0.05) | -0.01 (-0.02, 0.00) | 0.2702 |
| | Age 25yr (mmol/l) | -0.11 (-0.18, -0.03) | -0.04 (-0.08, -0.00) | -0.06 (-0.13, 0.00) | 0.0579 |
| HDL-c | Birth (mmol/l) | 0.58 (0.54, 0.61) | 0.50 (0.48, 0.52) | 0.08 (0.04, 0.11) | 0.0000 |
| | Change 0-7yr (mmol/l/yr) | 0.14 (0.13, 0.15) | 0.15 (0.15, 0.15) | -0.01 (-0.02, -0.00) | 0.0016 |
| | Change 7-18yr (mmol/l/yr) | -0.04 (-0.04, -0.03) | -0.04 (-0.04, -0.03) | -0.00 (-0.00, 0.00) | 0.7878 |
| | Change 18-25yr (mmol/l/yr) | 0.04 (0.03, 0.05) | 0.04 (0.03, 0.04) | 0.00 (-0.01, 0.01) | 0.9000 |
| | Age 25yr (mmol/l) | 1.41 (1.34, 1.47) | 1.40 (1.37, 1.44) | 0.00 (-0.05, 0.06) | 0.8890 |
| Non-HDL-c | Birth (mmol/l) | 1.41 (1.29, 1.53) | 1.36 (1.30, 1.42) | 0.05 (-0.06, 0.16) | 0.3366 |
| | Change 0-9yr (mmol/l/yr) | 0.17 (0.15, 0.18) | 0.18 (0.17, 0.18) | -0.01 (-0.03, 0.00) | 0.1865 |
| | Change 9-18yr (mmol/l/yr) | -0.07 (-0.09, -0.06) | -0.08 (-0.08, -0.07) | 0.00 (-0.01, 0.01) | 0.7920 |
| | Change 18-25yr (mmol/l/yr) | 0.12 (0.10, 0.14) | 0.11 (0.10, 0.12) | 0.01 (-0.01, 0.02) | 0.5630 |
| | Age 25yr (mmol/l) | 3.06 (2.92, 3.19) | 3.05 (2.97, 3.12) | 0.01 (-0.11, 0.13) | 0.8839 |

Predicted estimates based females, white ethnicity, mean maternal age and pre pregnancy BMI, no HDP, no previous children, no maternal smoking or alcohol, and educated to CSE/ Vocational/ O-level, no existing diabetes, GDM or glycosuria and no treatments to help conceive.

**Table S10 Intercept and slope estimates for glucose and insulin**

| | Age (years) | Preterm Mean (95% CI) | Term Mean (95% CI) | Mean difference (95% CI) comparing pre-term to term born participants | P value for difference |
|---|---|---|---|---|---|
| Glucose | 7 years | 4.23 (4.16, 4.30) | 4.25 (4.21, 4.29) | -0.02 (-0.08, 0.04) | 0.5342 |
| | Change 7-15 yr (mmol/l/yr) | 0.13 (0.12, 0.15) | 0.14 (0.13, 0.14) | -0.00 (-0.01, 0.01) | 0.7992 |
| | Change 15-18 yr (mmol/l/yr) | -0.04 (-0.08, -0.00) | -0.07 (-0.10, -0.05) | 0.03 (-0.00, 0.07) | 0.0786 |
| | Change 18-25yr (mmol//yr) | 0.05 (0.04, 0.07) | 0.05 (0.04, 0.06) | -0.00 (-0.02, 0.01) | 0.9179 |
| | Age 25yr (mmol/) | 5.55 (5.45, 5.66) | 5.49 (5.43, 5.55) | 0.06 (-0.03, 0.16) | 0.1996 |
| Insulin (logged) | Birth | 0.89 (0.67, 1.11) | 0.95 (0.84, 1.05) | -0.06 (-0.27, 0.16) | 0.5959 |
| | Change 0-9 yr (mmol/l/yr) | 0.05 (0.01, 0.09) | 0.06 (0.04, 0.08) | -0.01 (-0.05, 0.03) | 0.5956 |
| | Change 9-15 yr (mmol/l/yr) | 0.13 (0.08, 0.18) | 0.11 (0.09, 0.14) | 0.02 (-0.03, 0.06) | 0.4351 |
| | Change 15-18yr (mmol//yr) | -0.11 (-0.15, -0.06) | -0.11 (-0.14, -0.09) | 0.01 (-0.04, 0.05) | 0.7575 |
| | Change 18-25yr (mmol//yr) | 0.04 (0.01, 0.06) | 0.03 (0.02, 0.05) | 0.00 (-0.02, 0.02) | 0.9502 |
| | Age 25yr (mmol/) | 2.05 (1.93, 2.16) | 2.06 (2.00, 2.12) | -0.02 (-0.12, 0.08) | 0.7578 |

**Table S11 Predicted mean BMI (95% CI) at ages 1, 3, 7, 9, 11, 13, 15, 18 and 25 years by preterm and full-term birth and the difference in their means (95% CI) from sensitivity analyses**

| | | Preterm Mean (95% CI) | Term Mean (95% CI) | Mean difference (95% CI) comparing pre-term to term born participants | P value for difference |
|---|---|---|---|---|---|
| Main analysis (adjusted) | 1 | 2.73 (2.52, 2.95) | 2.73 (2.55, 2.92) | -0.00 (-0.12, 0.12) | 0.9748 |
| Complete case | 1 | 2.74 (2.50, 2.99) | 2.73 (2.53, 2.94) | 0.01 (-0.12, 0.14) | 0.8840 |
| Unadjusted | 1 | 2.78 (2.77, 2.80) | 2.82 (2.81, 2.82) | -0.03 (-0.05, -0.02) | 0.0002 |
| Including post terms | 1 | 2.69 (2.48, 2.90) | 2.69 (2.51, 2.87) | -0.00 (-0.12, 0.12) | 0.9957 |
| Main analysis (adjusted) | 3 | 2.70 (2.63, 2.77) | 2.71 (2.66, 2.77) | -0.01 (-0.06, 0.03) | 0.5771 |
| Complete case | 3 | 2.70 (2.62, 2.78) | 2.71 (2.65, 2.78) | -0.01 (-0.06, 0.04) | 0.7044 |
| Unadjusted | 3 | 2.76 (2.75, 2.77) | 2.78 (2.78, 2.78) | -0.02 (-0.04, -0.01) | 0.0001 |
| Including post terms | 3 | 2.69 (2.62, 2.76) | 2.70 (2.65, 2.76) | -0.01 (-0.06, 0.03) | 0.5753 |
| Main analysis (adjusted) | 7 | 2.75 (2.73, 2.77) | 2.77 (2.76, 2.78) | -0.02 (-0.03, -0.00) | 0.0416 |
| Complete case | 7 | 2.75 (2.73, 2.77) | 2.77 (2.76, 2.78) | -0.02 (-0.04, 0.00) | 0.0951 |
| Unadjusted | 7 | 2.79 (2.78, 2.81) | 2.81 (2.81, 2.82) | -0.02 (-0.03, -0.01) | 0.0018 |
| Including post terms | 7 | 2.75 (2.73, 2.77) | 2.77 (2.76, 2.78) | -0.02 (-0.03, -0.00) | 0.0353 |
| Main analysis (adjusted) | 9 | 2.81 (2.79, 2.82) | 2.82 (2.81, 2.83) | -0.02 (-0.03, -0.00) | 0.0099 |
| Complete case | 9 | 2.81 (2.79, 2.82) | 2.82 (2.81, 2.83) | -0.02 (-0.03, -0.00) | 0.0463 |
| Unadjusted | 9 | 2.84 (2.82, 2.85) | 2.85 (2.85, 2.86) | -0.02 (-0.03, -0.01) | 0.0031 |

| | | | | | |
|---|---|---|---|---|---|
| Including post terms | 9 | 2.81 (2.79, 2.82) | 2.82 (2.82, 2.83) | -0.02 (-0.03, -0.00) | 0.0079 |
| Main analysis (adjusted) | 11 | 2.87 (2.86, 2.88) | 2.89 (2.88, 2.89) | -0.02 (-0.03, -0.00) | 0.0062 |
| Complete case | 11 | 2.87 (2.86, 2.89) | 2.89 (2.88, 2.90) | -0.01 (-0.03, 0.00) | 0.0527 |
| Unadjusted | 11 | 2.89 (2.88, 2.90) | 2.91 (2.90, 2.91) | -0.02 (-0.03, -0.01) | 0.0051 |
| Including post terms | 11 | 2.87 (2.86, 2.88) | 2.89 (2.88, 2.89) | -0.02 (-0.03, -0.01) | 0.0051 |
| Main analysis (adjusted) | 13 | 2.94 (2.92, 2.95) | 2.95 (2.95, 2.96) | -0.02 (-0.03, -0.00) | 0.0090 |
| Complete case | 13 | 2.94 (2.93, 2.96) | 2.96 (2.95, 2.96) | -0.01 (-0.03, 0.00) | 0.0992 |
| Unadjusted | 13 | 2.95 (2.94, 2.96) | 2.97 (2.97, 2.97) | -0.02 (-0.03, -0.00) | 0.0100 |
| Including post terms | 13 | 2.94 (2.92, 2.95) | 2.95 (2.95, 2.96) | -0.02 (-0.03, -0.00) | 0.0081 |
| Main analysis (adjusted) | 15 | 3.00 (2.99, 3.01) | 3.01 (3.01, 3.02) | -0.02 (-0.03, -0.00) | 0.0201 |
| Complete case | 15 | 3.01 (2.99, 3.02) | 3.02 (3.01, 3.03) | -0.01 (-0.03, 0.01) | 0.2009 |
| Unadjusted | 15 | 3.02 (3.01, 3.03) | 3.03 (3.03, 3.04) | -0.02 (-0.03, -0.00) | 0.0236 |
| Including post terms | 15 | 3.00 (2.99, 3.01) | 3.01 (3.01, 3.02) | -0.01 (-0.03, -0.00) | 0.0197 |
| Main analysis (adjusted) | 18 | 3.06 (3.05, 3.08) | 3.08 (3.07, 3.09) | -0.01 (-0.03, 0.00) | 0.0731 |
| Complete case | 18 | 3.07 (3.05, 3.09) | 3.08 (3.07, 3.09) | -0.01 (-0.02, 0.01) | 0.4235 |
| Unadjusted | 18 | 3.12 (3.10, 3.13) | 3.13 (3.12, 3.13) | -0.01 (-0.03, 0.00) | 0.0995 |
| Including post terms | 18 | 3.07 (3.05, 3.08) | 3.08 (3.07, 3.09) | -0.01 (-0.03, 0.00) | 0.0769 |
| Main analysis (adjusted) | 25 | 2.87 (2.78, 2.95) | 2.87 (2.79, 2.96) | -0.01 (-0.03, 0.01) | 0.5466 |
| Complete case | 25 | 2.85 (2.75, 2.94) | 2.85 (2.76, 2.95) | -0.01 (-0.03, 0.01) | 0.4451 |
| Unadjusted | 25 | 3.21 (3.19, 3.22) | 3.20 (3.20, 3.20) | 0.01 (-0.01, 0.02) | 0.5393 |
| Including post terms | 25 | 2.90 (2.82, 2.98) | 2.90 (2.82, 2.98) | -0.01 (-0.03, 0.01) | 0.5157 |

Main analysis (as shown in Figure 1 and Table 3): main analysis of preterm (24-36 weeks) vs full term (37-41 weeks) adjusted for (and entered into the models as main effects and interactions with offspring age) offspring sex and maternal characteristics: age, parity, smoking status, alcohol intake, pre pregnancy BMI, HDP or pre-existing hypertension, existing diabetes or GDM or glycosuria, ethnicity (white compared to non-white) and any treatments to help conception in this pregnancy (including IVF) (compared to none). Predictions described are for offspring females, white ethnicity, mean maternal age and pre pregnancy BMI, no HDP or pre-existing hypertension, no previous children, no maternal smoking or alcohol, educated to CSE/ Vocational/ O-level, no existing diabetes, GDM or glycosuria and no treatments to help conceive. Estimates <0 mean lower values for preterm vs term. Complete case: only those with complete data included and adjusted for the same confounders as described above. Unadjusted associations based on the multiple imputation dataset to enable comparison of the same population to the main analysis model. All post-term births (i.e. women with gestation >41 weeks included) and adjusted for the same confounders as described above.

**Table S12 Predicted mean lean mass (95% CI) at ages 9, 11, 13, 15, 18 and 25 years by preterm and full-term birth and the difference in their means (95% CI) from sensitivity analyses**

| | | Preterm Mean (95% CI) | Term Mean (95% CI) | Mean difference (95% CI) comparing pre-term to term born participants | P value for difference |
|---|---|---|---|---|---|
| Main analysis (adjusted) | 9 | 22.8 (22.4, 23.1) | 23.3 (23.1, 23.5) | -0.6 (-0.9, -0.2) | 0.001 |
| Complete case | 9 | 22.8 (22.3, 23.3) | 23.3 (23.0, 23.5) | -0.5 (-0.9, -0.1) | 0.019 |
| Unadjusted | 9 | 21.5 (21.1, 22.0) | 21.9 (21.8, 22.0) | -0.3 (-0.8, 0.1) | 0.117 |
| Including post terms | 9 | 22.8 (22.4, 23.2) | 23.4 (23.1, 23.6) | -0.6 (-0.9, -0.2) | 0.001 |

| | | | | | |
|---|---|---|---|---|---|
| Main analysis (adjusted) | 11 | 27.2 (26.8, 27.6) | 27.9 (27.7, 28.1) | -0.7 (-1.1, -0.4) | 0.000 |
| Complete case | 11 | 27.3 (26.8, 27.7) | 27.9 (27.6, 28.1) | -0.6 (-1.0, -0.2) | 0.005 |
| Unadjusted | 11 | 27.1 (26.7, 27.5) | 27.8 (27.7, 27.9) | -0.7 (-1.1, -0.3) | 0.000 |
| Including post terms | 11 | 27.2 (26.8, 27.6) | 27.9 (27.7, 28.1) | -0.7 (-1.1, -0.4) | 0.000 |
| Main analysis (adjusted) | 13 | 35.1 (30.0, 40.3) | 32.5 (32.1, 32.8) | -0.9 (-1.4, -0.3) | 0.003 |
| Complete case | 13 | 35.8 (29.9, 41.8) | 32.5 (32.0, 32.9) | -0.7 (-1.4, -0.0) | 0.050 |
| Unadjusted | 13 | 45.6 (38.0, 53.2) | 33.8 (33.5, 34.0) | -1.1 (-2.0, -0.3) | 0.009 |
| Including post terms | 13 | 35.3 (30.2, 40.3) | 32.5 (32.1, 32.8) | -0.9 (-1.5, -0.3) | 0.002 |
| Main analysis (adjusted) | 15 | 47.0 (46.4, 47.7) | 47.5 (47.1, 47.8) | -0.5 (-1.1, 0.1) | 0.116 |
| Complete case | 15 | 45.9 (45.1, 46.7) | 46.0 (45.5, 46.5) | -0.1 (-0.8, 0.5) | 0.678 |
| Unadjusted | 15 | 43.0 (42.2, 43.8) | 42.5 (42.3, 42.7) | 0.5 (-0.4, 1.3) | 0.283 |
| Including post terms | 15 | 45.8 (45.1, 46.4) | 46.2 (45.8, 46.6) | -0.5 (-1.1, 0.1) | 0.099 |
| Main analysis (adjusted) | 18 | 54.6 (53.9, 55.2) | 55.3 (55.0, 55.7) | -0.8 (-1.4, -0.2) | 0.012 |
| Complete case | 18 | 54.2 (53.4, 55.0) | 54.9 (54.5, 55.4) | -0.7 (-1.4, -0.0) | 0.046 |
| Unadjusted | 18 | 46.3 (45.4, 47.2) | 45.8 (45.5, 46.0) | 0.5 (-0.4, 1.4) | 0.241 |
| Including post terms | 18 | 54.2 (53.5, 54.8) | 55.0 (54.7, 55.4) | -0.8 (-1.4, -0.2) | 0.006 |
| Main analysis (adjusted) | 25 | 55.1 (54.3, 55.8) | 56.3 (55.9, 56.7) | -1.3 (-1.9, -0.6) | 0.000 |
| Complete case | 25 | 34.4 (32.2, 36.5) | 35.2 (33.1, 37.3) | -0.8 (-1.6, -0.1) | 0.025 |
| Unadjusted | 25 | 47.5 (46.6, 48.4) | 47.9 (47.7, 48.1) | -0.4 (-1.4, 0.5) | 0.358 |
| Including post terms | 25 | 34.3 (32.5, 36.1) | 35.2 (33.5, 36.9) | -0.9 (-1.5, -0.3) | 0.003 |

**Table S13 Predicted mean (logged) fat mass (95% CI) at ages 9, 11, 13, 15, 18 and 25 years by preterm and full-term birth and the difference in their means (95% CI) from sensitivity analyses**

| | | Preterm Mean (95% CI) | Term Mean (95% CI) | Mean difference (95% CI) comparing pre-term to term born participants | P value for difference |
|---|---|---|---|---|---|
| Main analysis (adjusted) | 9 | 2.0 (1.9, 2.0) | 2.0 (2.0, 2.1) | -0.1 (-0.1, -0.0) | 0.006 |
| Complete case | 9 | 1.9 (1.8, 2.0) | 1.9 (1.9, 2.0) | -0.1 (-0.1, 0.0) | 0.082 |
| Unadjusted | 9 | 1.8 (1.7, 1.8) | 1.9 (1.8, 1.9) | -0.1 (-0.1, -0.0) | 0.007 |
| Including post terms | 9 | 1.8 (1.8, 1.9) | 1.9 (1.9, 2.0) | -0.1 (-0.1, -0.0) | 0.011 |
| Main analysis (adjusted) | 11 | 2.1 (2.0, 2.1) | 2.1 (2.1, 2.2) | -0.1 (-0.1, -0.0) | 0.004 |
| Complete case | 11 | 2.0 (1.9, 2.1) | 2.1 (2.0, 2.1) | -0.1 (-0.1, -0.0) | 0.017 |
| Unadjusted | 11 | 2.1 (2.0, 2.1) | 2.2 (2.2, 2.2) | -0.1 (-0.1, -0.0) | 0.001 |
| Including post terms | 11 | 2.0 (1.9, 2.1) | 2.1 (2.0, 2.1) | -0.1 (-0.1, -0.0) | 0.002 |
| Main analysis (adjusted) | 13 | 2.2 (1.9, 2.6) | 2.3 (2.2, 2.3) | -0.1 (-0.1, -0.0) | 0.016 |
| Complete case | 13 | 2.3 (1.8, 2.9) | 2.2 (2.2, 2.3) | -0.1 (-0.1, -0.0) | 0.022 |
| Unadjusted | 13 | 2.2 (1.7, 2.7) | 2.5 (2.5, 2.5) | -0.1 (-0.1, -0.0) | 0.006 |
| Including post terms | 13 | 2.2 (1.7, 2.7) | 2.2 (2.2, 2.3) | -0.1 (-0.1, -0.0) | 0.008 |
| Main analysis (adjusted) | 15 | 2.1 (2.0, 2.2) | 2.2 (2.1, 2.2) | -0.1 (-0.1, 0.0) | 0.058 |
| Complete case | 15 | 2.1 (2.1, 2.2) | 2.2 (2.1, 2.2) | -0.1 (-0.1, 0.0) | 0.119 |
| Unadjusted | 15 | 2.4 (2.3, 2.5) | 2.5 (2.5, 2.5) | -0.1 (-0.2, -0.1) | 0.000 |
| Including post terms | 15 | 2.1 (2.0, 2.2) | 2.2 (2.1, 2.2) | -0.1 (-0.1, -0.0) | 0.029 |
| Main analysis (adjusted) | 18 | 2.4 (2.3, 2.5) | 2.4 (2.4, 2.5) | -0.1 (-0.1, 0.0) | 0.052 |
| Complete case | 18 | 2.4 (2.3, 2.5) | 2.4 (2.3, 2.5) | -0.1 (-0.1, 0.0) | 0.076 |
| Unadjusted | 18 | 2.7 (2.6, 2.7) | 2.8 (2.7, 2.8) | -0.1 (-0.1, -0.0) | 0.007 |

| | | | | | |
|---|---|---|---|---|---|
| Including post terms | 18 | 2.3 (2.3, 2.4) | 2.4 (2.3, 2.5) | -0.1 (-0.1, 0.0) | 0.069 |
| Main analysis (adjusted) | 25 | 3.0 (2.9, 3.0) | 3.0 (3.0, 3.0) | -0.0 (-0.1, 0.0) | 0.657 |
| Complete case | 25 | 3.4 (2.5, 4.3) | 3.4 (2.5, 4.3) | 0.0 (-0.1, 0.1) | 0.943 |
| Unadjusted | 25 | 3.1 (3.0, 3.1) | 3.1 (3.0, 3.1) | 0.0 (-0.1, 0.1) | 0.809 |
| Including post terms | 25 | 3.9 (3.1, 4.6) | 3.9 (3.1, 4.6) | 0.0 (-0.1, 0.1) | 0.862 |

**Table S14 Predicted mean SBP (95% CI) at ages 7, 9, 11, 13, 15, 18 and 25 years by preterm and full-term birth and the difference in their means (95% CI) from sensitivity analyses**

| | | Preterm Mean (95% CI) | Term Mean (95% CI) | Mean difference (95% CI) comparing pre-term to term born participants | P value for difference |
|---|---|---|---|---|---|
| Main analysis (adjusted) | 7 | 99.4 (98.5, 100.4) | 98.7 (98.2, 99.3) | 0.7 (-0.2, 1.6) | 0.117 |
| Complete case | 7 | 99.2 (98.0, 100.3) | 98.8 (98.1, 99.4) | 0.4 (-0.7, 1.5) | 0.464 |
| Unadjusted | 7 | 99.5 (98.6, 100.4) | 98.5 (98.2, 98.7) | 1.1 (0.2, 2.0) | 0.020 |
| Including post terms | 7 | 99.5 (98.5, 100.5) | 98.6 (98.1, 99.2) | 0.8 (-0.1, 1.7) | 0.065 |
| Main analysis (adjusted) | 9 | 102.1 (101.4, 102.9) | 101.1 (100.7, 101.5) | 1.0 (0.3, 1.7) | 0.004 |
| Complete case | 9 | 101.8 (100.9, 102.7) | 101.1 (100.7, 101.6) | 0.6 (-0.2, 1.5) | 0.123 |
| Unadjusted | 9 | 102.7 (102.0, 103.3) | 101.4 (101.3, 101.6) | 1.3 (0.6, 1.9) | 0.000 |
| Including post terms | 9 | 102.2 (101.4, 102.9) | 101.1 (100.7, 101.4) | 1.1 (0.4, 1.8) | 0.001 |
| Main analysis (adjusted) | 11 | 104.8 (104.0, 105.6) | 103.5 (103.1, 103.9) | 1.3 (0.6, 2.0) | 0.000 |
| Complete case | 11 | 104.4 (103.5, 105.3) | 103.5 (103.0, 104.0) | 0.9 (0.0, 1.7) | 0.042 |
| Unadjusted | 11 | 105.9 (105.2, 106.6) | 104.4 (104.2, 104.6) | 1.5 (0.8, 2.2) | 0.000 |
| Including post terms | 11 | 104.8 (104.1, 105.6) | 103.5 (103.1, 103.9) | 1.4 (0.7, 2.1) | 0.000 |
| Main analysis (adjusted) | 13 | 111.1 (110.3, 111.9) | 109.9 (109.4, 110.3) | 1.2 (0.5, 2.0) | 0.001 |
| Complete case | 13 | 110.8 (109.9, 111.8) | 109.9 (109.4, 110.4) | 0.9 (0.1, 1.8) | 0.036 |
| Unadjusted | 13 | 111.5 (110.8, 112.2) | 110.0 (109.8, 110.1) | 1.6 (0.9, 2.3) | 0.000 |
| Including post terms | 13 | 111.2 (110.4, 111.9) | 109.8 (109.4, 110.2) | 1.3 (0.6, 2.0) | 0.000 |
| Main analysis (adjusted) | 15 | 121.1 (120.0, 122.2) | 120.1 (119.5, 120.7) | 0.9 (-0.1, 1.9) | 0.072 |
| Complete case | 15 | 121.1 (119.7, 122.4) | 120.3 (119.6, 121.0) | 0.8 (-0.4, 2.0) | 0.208 |
| Unadjusted | 15 | 119.7 (118.7, 120.7) | 118.1 (117.9, 118.4) | 1.6 (0.6, 2.6) | 0.003 |
| Including post terms | 15 | 121.2 (120.1, 122.3) | 120.2 (119.6, 120.7) | 1.0 (0.0, 2.0) | 0.049 |
| Main analysis (adjusted) | 18 | 123.5 (122.2, 124.8) | 121.5 (120.8, 122.2) | 1.9 (0.8, 3.1) | 0.001 |
| Complete case | 18 | 122.6 (121.0, 124.1) | 121.4 (120.6, 122.2) | 1.2 (-0.2, 2.6) | 0.105 |
| Unadjusted | 18 | 118.8 (117.5, 120.1) | 115.7 (115.4, 116.0) | 3.1 (1.7, 4.5) | 0.000 |
| Including post terms | 18 | 123.5 (122.2, 124.8) | 121.4 (120.7, 122.0) | 2.1 (0.9, 3.3) | 0.001 |
| Main analysis (adjusted) | 25 | 123.7 (122.1, 125.2) | 122.8 (121.9, 123.6) | 0.9 (-0.5, 2.3) | 0.202 |
| Complete case | 25 | 123.6 (121.8, 125.5) | 123.1 (122.1, 124.1) | 0.6 (-1.1, 2.2) | 0.511 |
| Unadjusted | 25 | 118.0 (116.5, 119.5) | 116.3 (115.9, 116.6) | 1.7 (0.2, 3.3) | 0.026 |
| Including post terms | 25 | 123.7 (122.2, 125.3) | 122.9 (122.0, 123.7) | 0.9 (-0.5, 2.2) | 0.198 |

**Table S15 Predicted mean DBP (95% CI) at ages 7, 9, 11, 13, 15, 18 and 25 years by preterm and full-term birth and the difference in their means (95% CI) from sensitivity analyses**

| | | Preterm Mean (95% CI) | Term Mean (95% CI) | Mean difference (95% CI) comparing pre-term to term born participants | P value for difference |
|---|---|---|---|---|---|
| Main analysis (adjusted) | 7 | 57.7 (57.0, 58.4) | 57.1 (56.7, 57.5) | 0.6 (-0.0, 1.3) | 0.066 |
| Complete case | 7 | 57.3 (56.5, 58.2) | 57.0 (56.6, 57.5) | 0.3 (-0.5, 1.1) | 0.416 |
| Unadjusted | 7 | 58.0 (57.4, 58.6) | 57.3 (57.1, 57.4) | 0.8 (0.1, 1.4) | 0.024 |
| Including post terms | 7 | 57.7 (57.0, 58.5) | 57.1 (56.7, 57.4) | 0.7 (0.0, 1.3) | 0.045 |
| Main analysis (adjusted) | 9 | 57.6 (57.0, 58.1) | 57.2 (56.9, 57.5) | 0.4 (-0.1, 0.9) | 0.101 |
| Complete case | 9 | 57.2 (56.6, 57.8) | 57.2 (56.8, 57.5) | 0.0 (-0.5, 0.6) | 0.892 |
| Unadjusted | 9 | 58.0 (57.5, 58.4) | 57.4 (57.3, 57.6) | 0.5 (0.0, 1.0) | 0.031 |
| Including post terms | 9 | 57.6 (57.1, 58.1) | 57.1 (56.9, 57.4) | 0.5 (-0.0, 0.9) | 0.062 |
| Main analysis (adjusted) | 11 | 57.4 (56.9, 58.0) | 57.3 (56.9, 57.6) | 0.2 (-0.3, 0.7) | 0.479 |
| Complete case | 11 | 57.1 (56.4, 57.7) | 57.3 (57.0, 57.7) | -0.2 (-0.9, 0.4) | 0.443 |
| Unadjusted | 11 | 57.9 (57.4, 58.4) | 57.6 (57.5, 57.7) | 0.3 (-0.2, 0.8) | 0.240 |
| Including post terms | 11 | 57.4 (56.9, 58.0) | 57.2 (56.9, 57.5) | 0.2 (-0.3, 0.7) | 0.353 |
| Main analysis (adjusted) | 13 | 59.5 (58.9, 60.0) | 59.6 (59.3, 59.9) | -0.1 (-0.6, 0.4) | 0.657 |
| Complete case | 13 | 59.1 (58.4, 59.7) | 59.6 (59.2, 60.0) | -0.5 (-1.1, 0.1) | 0.089 |
| Unadjusted | 13 | 59.7 (59.2, 60.2) | 59.7 (59.5, 59.8) | 0.0 (-0.5, 0.5) | 0.870 |
| Including post terms | 13 | 59.5 (58.9, 60.0) | 59.6 (59.3, 59.8) | -0.1 (-0.6, 0.4) | 0.773 |
| Main analysis (adjusted) | 15 | 63.7 (62.8, 64.6) | 64.2 (63.7, 64.7) | -0.5 (-1.3, 0.3) | 0.224 |
| Complete case | 15 | 63.2 (62.2, 64.3) | 64.1 (63.5, 64.6) | -0.8 (-1.8, 0.1) | 0.088 |
| Unadjusted | 15 | 63.3 (62.5, 64.1) | 63.6 (63.4, 63.8) | -0.3 (-1.1, 0.5) | 0.518 |
| Including post terms | 15 | 63.7 (62.8, 64.6) | 64.2 (63.7, 64.6) | -0.5 (-1.3, 0.3) | 0.228 |
| Main analysis (adjusted) | 18 | 63.5 (62.5, 64.4) | 63.3 (62.8, 63.8) | 0.1 (-0.7, 1.0) | 0.758 |
| Complete case | 18 | 63.0 (61.9, 64.2) | 63.3 (62.7, 63.9) | -0.2 (-1.3, 0.8) | 0.641 |
| Unadjusted | 18 | 64.4 (63.6, 65.2) | 64.2 (64.0, 64.4) | 0.2 (-0.7, 1.0) | 0.708 |
| Including post terms | 18 | 63.5 (62.5, 64.4) | 63.2 (62.8, 63.7) | 0.2 (-0.6, 1.1) | 0.614 |
| Main analysis (adjusted) | 25 | 68.1 (66.8, 69.4) | 68.4 (67.7, 69.1) | -0.3 (-1.5, 0.8) | 0.549 |
| Complete case | 25 | 67.8 (66.3, 69.3) | 68.7 (67.9, 69.5) | -0.9 (-2.3, 0.4) | 0.177 |
| Unadjusted | 25 | 67.0 (65.9, 68.1) | 67.1 (66.9, 67.4) | -0.1 (-1.2, 1.0) | 0.857 |
| Including post terms | 25 | 68.2 (66.9, 69.4) | 68.5 (67.8, 69.2) | -0.3 (-1.4, 0.8) | 0.573 |

**Table S16 Predicted mean pulse rate (95% CI) at ages 7, 9, 11, 13, 15, 18 and 25 years by preterm and full-term birth and the difference in their means (95% CI) from sensitivity analyses**

| | | Preterm Mean (95% CI) | Term Mean (95% CI) | Mean difference (95% CI) comparing pre-term to term born participants | P value for difference |
|---|---|---|---|---|---|
| Main analysis (adjusted) | 7 | 83.2 (82.0, 84.4) | 83.0 (82.3, 83.6) | 0.2 (-0.8, 1.3) | 0.685 |
| Complete case | 7 | 82.5 (81.1, 83.8) | 82.8 (82.1, 83.6) | -0.4 (-1.6, 0.9) | 0.563 |
| Unadjusted | 7 | 84.3 (83.3, 85.4) | 84.0 (83.7, 84.3) | 0.3 (-0.7, 1.4) | 0.559 |

| | | | | | |
|---|---|---|---|---|---|
| Including post terms | 7 | 83.1 (82.0, 84.3) | 82.9 (82.2, 83.5) | 0.3 (-0.8, 1.3) | 0.615 |
| Main analysis (adjusted) | 9 | 78.3 (77.4, 79.2) | 78.3 (77.8, 78.8) | -0.0 (-0.8, 0.8) | 0.988 |
| Complete case | 9 | 77.6 (76.6, 78.7) | 78.3 (77.7, 78.8) | -0.6 (-1.6, 0.3) | 0.195 |
| Unadjusted | 9 | 79.7 (78.9, 80.5) | 79.7 (79.5, 79.9) | -0.0 (-0.8, 0.8) | 0.988 |
| Including post terms | 9 | 78.3 (77.5, 79.2) | 78.3 (77.8, 78.7) | 0.0 (-0.7, 0.8) | 0.913 |
| Main analysis (adjusted) | 11 | 73.4 (72.5, 74.2) | 73.6 (73.1, 74.1) | -0.2 (-1.0, 0.6) | 0.570 |
| Complete case | 11 | 72.8 (71.8, 73.9) | 73.7 (73.1, 74.3) | -0.9 (-1.9, 0.1) | 0.074 |
| Unadjusted | 11 | 75.1 (74.3, 75.8) | 75.4 (75.2, 75.6) | -0.3 (-1.1, 0.5) | 0.426 |
| Including post terms | 11 | 73.5 (72.6, 74.4) | 73.7 (73.2, 74.2) | -0.2 (-1.0, 0.6) | 0.650 |
| Main analysis (adjusted) | 13 | 70.6 (69.7, 71.5) | 71.1 (70.6, 71.6) | -0.5 (-1.3, 0.3) | 0.216 |
| Complete case | 13 | 70.3 (69.2, 71.4) | 71.4 (70.8, 72.0) | -1.1 (-2.1, -0.1) | 0.032 |
| Unadjusted | 13 | 72.7 (71.9, 73.5) | 73.4 (73.1, 73.6) | -0.7 (-1.5, 0.1) | 0.099 |
| Including post terms | 13 | 70.9 (70.0, 71.7) | 71.3 (70.8, 71.8) | -0.5 (-1.3, 0.3) | 0.261 |
| Main analysis (adjusted) | 15 | 70.1 (68.8, 71.3) | 70.9 (70.3, 71.6) | -0.9 (-2.0, 0.3) | 0.140 |
| Complete case | 15 | 70.1 (68.6, 71.6) | 71.3 (70.5, 72.1) | -1.2 (-2.6, 0.2) | 0.082 |
| Unadjusted | 15 | 72.5 (71.4, 73.6) | 73.6 (73.3, 73.9) | -1.1 (-2.3, 0.0) | 0.057 |
| Including post terms | 15 | 70.4 (69.1, 71.6) | 71.2 (70.5, 71.8) | -0.8 (-1.9, 0.3) | 0.163 |
| Main analysis (adjusted) | 18 | 62.9 (61.5, 64.4) | 61.8 (61.0, 62.6) | 1.1 (-0.2, 2.4) | 0.100 |
| Complete case | 18 | 61.6 (59.9, 63.4) | 61.6 (60.7, 62.5) | -0.0 (-1.6, 1.6) | 0.996 |
| Unadjusted | 18 | 64.8 (63.5, 66.1) | 63.9 (63.6, 64.3) | 0.9 (-0.4, 2.2) | 0.184 |
| Including post terms | 18 | 63.0 (61.5, 64.5) | 61.8 (61.0, 62.6) | 1.2 (-0.1, 2.5) | 0.074 |
| Main analysis (adjusted) | 25 | 64.9 (63.3, 66.5) | 64.8 (63.9, 65.6) | 0.1 (-1.3, 1.5) | 0.857 |
| Complete case | 25 | 65.3 (63.4, 67.1) | 65.7 (64.7, 66.7) | -0.4 (-2.1, 1.3) | 0.635 |
| Unadjusted | 25 | 67.2 (65.8, 68.5) | 67.1 (66.7, 67.4) | 0.1 (-1.3, 1.5) | 0.870 |
| Including post terms | 25 | 65.0 (63.4, 66.5) | 64.7 (63.9, 65.6) | 0.3 (-1.1, 1.6) | 0.710 |

**Table S17 Predicted mean (logged) triglycerides (95% CI) at birth, 7, 9, 15, 18 and 25 years by preterm and full-term birth and the difference in their means (95% CI) from sensitivity analyses**

| | | Preterm Mean (95% CI) | Term Mean (95% CI) | Mean difference (95% CI) comparing pre-term to term born participants | P value for difference |
|---|---|---|---|---|---|
| Main analysis (adjusted) | 0 | -0.8 (-0.9, -0.8) | -0.6 (-0.6, -0.6) | -0.2 (-0.3, -0.2) | 0.000 |
| Complete case | 0 | -0.8 (-0.9, -0.7) | -0.6 (-0.6, -0.6) | -0.2 (-0.3, -0.2) | 0.000 |
| Unadjusted | 0 | -0.9 (-0.9, -0.8) | -0.7 (-0.7, -0.7) | -0.2 (-0.3, -0.1) | 0.000 |
| Including post terms | 0 | -0.8 (-0.9, -0.8) | -0.6 (-0.6, -0.6) | -0.2 (-0.3, -0.2) | 0.000 |
| Main analysis (adjusted) | 7 | -0.2 (-0.2, -0.1) | -0.1 (-0.1, -0.1) | -0.1 (-0.1, -0.0) | 0.008 |
| Complete case | 7 | -0.1 (-0.2, -0.1) | -0.1 (-0.1, -0.1) | -0.0 (-0.1, 0.0) | 0.118 |
| Unadjusted | 7 | -0.1 (-0.2, -0.1) | -0.1 (-0.1, -0.1) | -0.1 (-0.1, -0.0) | 0.008 |
| Including post terms | 7 | -0.2 (-0.2, -0.1) | -0.1 (-0.1, -0.1) | -0.1 (-0.1, -0.0) | 0.005 |
| Main analysis (adjusted) | 9 | 0.0 (-0.0, 0.1) | 0.0 (0.0, 0.1) | -0.0 (-0.0, 0.0) | 0.904 |
| Complete case | 9 | 0.1 (0.0, 0.1) | 0.0 (0.0, 0.1) | 0.0 (-0.0, 0.1) | 0.495 |
| Unadjusted | 9 | 0.1 (0.0, 0.1) | 0.1 (0.1, 0.1) | -0.0 (-0.1, 0.0) | 0.788 |
| Including post terms | 9 | 0.0 (-0.0, 0.1) | 0.0 (0.0, 0.1) | -0.0 (-0.1, 0.0) | 0.876 |
| Main analysis (adjusted) | 15 | -0.2 (-0.3, -0.2) | -0.2 (-0.2, -0.2) | -0.0 (-0.1, 0.0) | 0.427 |
| Complete case | 15 | -0.2 (-0.2, -0.1) | -0.2 (-0.2, -0.2) | 0.0 (-0.0, 0.0) | 0.898 |

| | | | | | |
|---|---|---|---|---|---|
| Unadjusted | 15 | -0.2 (-0.2, -0.2) | -0.2 (-0.2, -0.2) | -0.0 (-0.1, 0.0) | 0.341 |
| Including post terms | 15 | -0.2 (-0.3, -0.2) | -0.2 (-0.2, -0.2) | -0.0 (-0.1, 0.0) | 0.431 |
| Main analysis (adjusted) | 18 | -0.3 (-0.4, -0.3) | -0.3 (-0.3, -0.3) | -0.0 (-0.1, 0.0) | 0.403 |
| Complete case | 18 | -0.3 (-0.4, -0.2) | -0.3 (-0.3, -0.3) | -0.0 (-0.1, 0.1) | 0.867 |
| Unadjusted | 18 | -0.3 (-0.4, -0.3) | -0.3 (-0.3, -0.3) | -0.0 (-0.1, 0.0) | 0.342 |
| Including post terms | 18 | -0.3 (-0.4, -0.3) | -0.3 (-0.3, -0.3) | -0.0 (-0.1, 0.0) | 0.415 |
| Main analysis (adjusted) | 25 | -0.1 (-0.2, -0.0) | -0.0 (-0.1, -0.0) | -0.1 (-0.1, 0.0) | 0.058 |
| Complete case | 25 | -0.1 (-0.2, 0.0) | -0.0 (-0.1, 0.0) | -0.0 (-0.1, 0.0) | 0.255 |
| Unadjusted | 25 | -0.2 (-0.2, -0.1) | -0.1 (-0.1, -0.1) | -0.0 (-0.1, 0.0) | 0.137 |
| Including post terms | 25 | -0.1 (-0.2, -0.0) | -0.0 (-0.1, 0.0) | -0.1 (-0.1, 0.0) | 0.063 |

**Table S18 Predicted mean HDL-c (95% CI) at birth, 7, 9, 15, 18 and 25 years by preterm and full-term birth and the difference in their means (95% CI) from sensitivity analyses**

| | | Preterm Mean (95% CI) | Term Mean (95% CI) | Mean difference (95% CI) comparing pre-term to term born participants | P value for difference |
|---|---|---|---|---|---|
| Main analysis (adjusted) | 0 | 0.6 (0.5, 0.6) | 0.5 (0.5, 0.5) | 0.1 (0.0, 0.1) | 0.000 |
| Complete case | 0 | 0.6 (0.6, 0.7) | 0.5 (0.5, 0.5) | 0.1 (0.1, 0.1) | 0.000 |
| Unadjusted | 0 | 0.6 (0.6, 0.6) | 0.5 (0.5, 0.5) | 0.1 (0.0, 0.1) | 0.000 |
| Including post terms | 0 | 0.6 (0.5, 0.6) | 0.5 (0.5, 0.5) | 0.1 (0.0, 0.1) | 0.000 |
| Main analysis (adjusted) | 7 | 1.6 (1.5, 1.6) | 1.5 (1.5, 1.6) | 0.0 (-0.0, 0.0) | 0.712 |
| Complete case | 7 | 1.6 (1.5, 1.6) | 1.5 (1.5, 1.6) | 0.0 (-0.0, 0.1) | 0.365 |
| Unadjusted | 7 | 1.5 (1.5, 1.5) | 1.5 (1.5, 1.5) | 0.0 (-0.0, 0.0) | 0.652 |
| Including post terms | 7 | 1.6 (1.5, 1.6) | 1.5 (1.5, 1.6) | 0.0 (-0.0, 0.0) | 0.622 |
| Main analysis (adjusted) | 9 | 1.5 (1.4, 1.5) | 1.5 (1.5, 1.5) | 0.0 (-0.0, 0.0) | 0.730 |
| Complete case | 9 | 1.5 (1.4, 1.5) | 1.5 (1.5, 1.5) | 0.0 (-0.0, 0.0) | 0.507 |
| Unadjusted | 9 | 1.5 (1.4, 1.5) | 1.5 (1.5, 1.5) | 0.0 (-0.0, 0.0) | 0.822 |
| Including post terms | 9 | 1.5 (1.4, 1.5) | 1.5 (1.5, 1.5) | 0.0 (-0.0, 0.0) | 0.616 |
| Main analysis (adjusted) | 15 | 1.3 (1.2, 1.3) | 1.3 (1.2, 1.3) | 0.0 (-0.0, 0.0) | 0.890 |
| Complete case | 15 | 1.2 (1.2, 1.3) | 1.3 (1.2, 1.3) | -0.0 (-0.0, 0.0) | 0.715 |
| Unadjusted | 15 | 1.3 (1.3, 1.3) | 1.3 (1.3, 1.3) | -0.0 (-0.0, 0.0) | 0.545 |
| Including post terms | 15 | 1.3 (1.2, 1.3) | 1.3 (1.2, 1.3) | 0.0 (-0.0, 0.0) | 0.729 |
| Main analysis (adjusted) | 18 | 1.1 (1.1, 1.2) | 1.1 (1.1, 1.2) | 0.0 (-0.0, 0.0) | 0.975 |
| Complete case | 18 | 1.1 (1.1, 1.2) | 1.1 (1.1, 1.2) | -0.0 (-0.1, 0.0) | 0.461 |
| Unadjusted | 18 | 1.2 (1.2, 1.3) | 1.2 (1.2, 1.2) | -0.0 (-0.1, 0.0) | 0.406 |
| Including post terms | 18 | 1.1 (1.1, 1.2) | 1.1 (1.1, 1.2) | 0.0 (-0.0, 0.0) | 0.821 |
| Main analysis (adjusted) | 25 | 1.4 (1.3, 1.5) | 1.4 (1.4, 1.4) | 0.0 (-0.1, 0.1) | 0.889 |
| Complete case | 25 | 1.4 (1.4, 1.5) | 1.4 (1.4, 1.5) | 0.0 (-0.0, 0.1) | 0.524 |
| Unadjusted | 25 | 1.6 (1.5, 1.6) | 1.6 (1.6, 1.6) | -0.0 (-0.1, 0.0) | 0.674 |
| Including post terms | 25 | 1.4 (1.3, 1.5) | 1.4 (1.4, 1.4) | 0.0 (-0.1, 0.1) | 0.826 |

**Table S19 Predicted mean non-HDL-c (95% CI) at birth, 7, 9, 15, 18 and 25 years by preterm and full-term birth and the difference in their means (95% CI) from sensitivity analyses**

| | | Preterm Mean (95% CI) | Term Mean (95% CI) | Mean difference (95% CI) | P value for difference |
|---|---|---|---|---|---|

| | | | | comparing pre-term to term born participants | |
|---|---|---|---|---|---|
| Main analysis (adjusted) | 0 | 1.4 (1.3, 1.5) | 1.4 (1.3, 1.4) | 0.1 (-0.1, 0.2) | 0.337 |
| Complete case | 0 | 1.4 (1.3, 1.6) | 1.4 (1.3, 1.5) | 0.1 (-0.1, 0.2) | 0.478 |
| Unadjusted | 0 | 1.4 (1.3, 1.5) | 1.4 (1.3, 1.4) | 0.1 (-0.1, 0.2) | 0.311 |
| Including post terms | 0 | 1.4 (1.3, 1.5) | 1.4 (1.3, 1.4) | 0.1 (-0.1, 0.2) | 0.374 |
| Main analysis (adjusted) | 7 | 2.6 (2.5, 2.6) | 2.6 (2.6, 2.6) | -0.0 (-0.1, 0.0) | 0.530 |
| Complete case | 7 | 2.6 (2.5, 2.7) | 2.6 (2.5, 2.6) | 0.0 (-0.0, 0.1) | 0.475 |
| Unadjusted | 7 | 2.6 (2.6, 2.7) | 2.7 (2.7, 2.7) | -0.0 (-0.1, 0.0) | 0.395 |
| Including post terms | 7 | 2.6 (2.5, 2.6) | 2.6 (2.6, 2.6) | -0.0 (-0.1, 0.0) | 0.504 |
| Main analysis (adjusted) | 9 | 2.4 (2.4, 2.5) | 2.4 (2.4, 2.5) | -0.0 (-0.1, 0.0) | 0.555 |
| Complete case | 9 | 2.5 (2.4, 2.5) | 2.4 (2.4, 2.5) | 0.0 (-0.0, 0.1) | 0.419 |
| Unadjusted | 9 | 2.5 (2.5, 2.6) | 2.5 (2.5, 2.5) | -0.0 (-0.1, 0.0) | 0.340 |
| Including post terms | 9 | 2.4 (2.4, 2.5) | 2.5 (2.4, 2.5) | -0.0 (-0.1, 0.0) | 0.519 |
| Main analysis (adjusted) | 15 | 2.0 (1.9, 2.1) | 2.0 (1.9, 2.0) | -0.0 (-0.1, 0.1) | 0.839 |
| Complete case | 15 | 2.0 (1.9, 2.1) | 2.0 (1.9, 2.0) | 0.0 (-0.1, 0.1) | 0.529 |
| Unadjusted | 15 | 2.1 (2.0, 2.1) | 2.1 (2.1, 2.1) | -0.0 (-0.1, 0.0) | 0.471 |
| Including post terms | 15 | 2.0 (1.9, 2.1) | 2.0 (2.0, 2.0) | -0.0 (-0.1, 0.1) | 0.789 |
| Main analysis (adjusted) | 18 | 1.8 (1.6, 1.9) | 1.8 (1.7, 1.8) | -0.0 (-0.1, 0.1) | 0.939 |
| Complete case | 18 | 1.8 (1.7, 1.9) | 1.8 (1.7, 1.8) | 0.0 (-0.1, 0.1) | 0.608 |
| Unadjusted | 18 | 1.8 (1.7, 1.9) | 1.9 (1.8, 1.9) | -0.0 (-0.1, 0.1) | 0.567 |
| Including post terms | 18 | 1.8 (1.7, 1.9) | 1.8 (1.7, 1.8) | -0.0 (-0.1, 0.1) | 0.892 |
| Main analysis (adjusted) | 25 | 2.6 (2.4, 2.7) | 2.5 (2.5, 2.6) | 0.0 (-0.1, 0.2) | 0.612 |
| Complete case | 25 | 2.6 (2.5, 2.8) | 2.6 (2.5, 2.7) | 0.0 (-0.1, 0.2) | 0.739 |
| Unadjusted | 25 | 2.5 (2.3, 2.6) | 2.4 (2.4, 2.4) | 0.0 (-0.1, 0.2) | 0.511 |
| Including post terms | 25 | 2.6 (2.5, 2.7) | 2.6 (2.5, 2.6) | 0.0 (-0.1, 0.2) | 0.679 |

**Table S20 Predicted mean glucose (95% CI) at ages 7, 9, 15, 18 and 25 years by preterm and full-term birth and the difference in their means (95% CI) from sensitivity analyses**

| | | Preterm Mean (95% CI) | Term Mean (95% CI) | Mean difference (95% CI) comparing pre-term to term born participants | P value for difference |
|---|---|---|---|---|---|
| Main analysis (adjusted) | 7 | 4.2 (4.2, 4.3) | 4.3 (4.2, 4.3) | -0.0 (-0.1, 0.0) | 0.534 |
| Complete case | 7 | 4.2 (4.2, 4.3) | 4.2 (4.2, 4.3) | -0.0 (-0.1, 0.1) | 0.722 |
| Unadjusted | 7 | 4.2 (4.1, 4.2) | 4.2 (4.2, 4.2) | -0.0 (-0.1, 0.0) | 0.598 |
| Including post terms | 7 | 4.2 (4.2, 4.3) | 4.2 (4.2, 4.3) | -0.0 (-0.1, 0.0) | 0.543 |
| Main analysis (adjusted) | 9 | 4.5 (4.4, 4.6) | 4.5 (4.5, 4.6) | -0.0 (-0.1, 0.0) | 0.370 |
| Complete case | 9 | 4.5 (4.4, 4.6) | 4.5 (4.5, 4.6) | -0.0 (-0.1, 0.0) | 0.693 |
| Unadjusted | 9 | 4.4 (4.4, 4.5) | 4.4 (4.4, 4.5) | -0.0 (-0.1, 0.0) | 0.589 |
| Including post terms | 9 | 4.5 (4.4, 4.6) | 4.5 (4.5, 4.5) | -0.0 (-0.1, 0.0) | 0.347 |
| Main analysis (adjusted) | 15 | 5.3 (5.2, 5.4) | 5.3 (5.3, 5.4) | -0.0 (-0.1, 0.0) | 0.354 |
| Complete case | 15 | 5.3 (5.2, 5.4) | 5.3 (5.3, 5.4) | -0.0 (-0.1, 0.1) | 0.861 |
| Unadjusted | 15 | 5.3 (5.2, 5.3) | 5.3 (5.3, 5.3) | -0.0 (-0.1, 0.1) | 0.894 |
| Including post terms | 15 | 5.3 (5.2, 5.4) | 5.3 (5.3, 5.4) | -0.0 (-0.1, 0.0) | 0.279 |
| Main analysis (adjusted) | 18 | 5.2 (5.1, 5.3) | 5.1 (5.1, 5.2) | 0.1 (-0.0, 0.2) | 0.121 |

| | | | | | |
|---|---|---|---|---|---|
| Complete case | 18 | 5.2 (5.1, 5.3) | 5.1 (5.1, 5.2) | 0.0 (-0.1, 0.1) | 0.355 |
| Unadjusted | 18 | 5.1 (5.0, 5.2) | 5.0 (5.0, 5.0) | 0.1 (-0.0, 0.2) | 0.079 |
| Including post terms | 18 | 5.2 (5.1, 5.3) | 5.1 (5.1, 5.2) | 0.1 (-0.0, 0.2) | 0.149 |
| Main analysis (adjusted) | 25 | 5.6 (5.4, 5.7) | 5.5 (5.4, 5.6) | 0.1 (-0.0, 0.2) | 0.200 |
| Complete case | 25 | 5.6 (5.5, 5.7) | 5.5 (5.4, 5.6) | 0.1 (-0.0, 0.2) | 0.099 |
| Unadjusted | 25 | 5.4 (5.3, 5.5) | 5.3 (5.3, 5.4) | 0.1 (-0.0, 0.2) | 0.179 |
| Including post terms | 25 | 5.6 (5.4, 5.7) | 5.5 (5.4, 5.6) | 0.1 (-0.0, 0.2) | 0.258 |

**Table S21 Predicted mean (logged) insulin (95% CI) at birth, 9, 15, 18 and 25 years by preterm and full-term birth and the difference in their means (95% CI) from sensitivity analyses**

| | | Preterm Mean (95% CI) | Term Mean (95% CI) | Mean difference (95% CI) comparing pre-term to term born participants | P value for difference |
|---|---|---|---|---|---|
| Main analysis (adjusted) | 0 | 0.9 (0.7, 1.1) | 0.9 (0.8, 1.1) | -0.1 (-0.3, 0.2) | 0.596 |
| Complete case | 0 | 0.9 (0.6, 1.1) | 0.9 (0.8, 1.1) | -0.1 (-0.3, 0.2) | 0.646 |
| Unadjusted | 0 | 1.0 (0.8, 1.2) | 1.1 (1.1, 1.2) | -0.1 (-0.3, 0.1) | 0.468 |
| Including post terms | 0 | 0.9 (0.7, 1.1) | 0.9 (0.8, 1.0) | -0.0 (-0.3, 0.2) | 0.717 |
| Main analysis (adjusted) | 9 | 1.3 (1.0, 1.6) | 1.5 (1.3, 1.6) | -0.2 (-0.4, 0.1) | 0.259 |
| Complete case | 9 | 1.4 (1.1, 1.7) | 1.4 (1.3, 1.6) | -0.0 (-0.3, 0.3) | 0.843 |
| Unadjusted | 9 | 1.4 (1.1, 1.6) | 1.5 (1.5, 1.6) | -0.2 (-0.4, 0.1) | 0.213 |
| Including post terms | 9 | 1.3 (1.1, 1.6) | 1.5 (1.4, 1.6) | -0.2 (-0.4, 0.1) | 0.238 |
| Main analysis (adjusted) | 15 | 2.1 (2.0, 2.2) | 2.2 (2.1, 2.2) | -0.0 (-0.1, 0.0) | 0.344 |
| Complete case | 15 | 2.1 (2.0, 2.2) | 2.2 (2.1, 2.2) | -0.0 (-0.1, 0.1) | 0.829 |
| Unadjusted | 15 | 2.2 (2.1, 2.3) | 2.2 (2.2, 2.3) | -0.1 (-0.2, 0.0) | 0.096 |
| Including post terms | 15 | 2.1 (2.0, 2.2) | 2.2 (2.1, 2.2) | -0.0 (-0.1, 0.0) | 0.344 |
| Main analysis (adjusted) | 18 | 1.8 (1.7, 1.9) | 1.8 (1.8, 1.9) | -0.0 (-0.1, 0.1) | 0.686 |
| Complete case | 18 | 1.8 (1.7, 1.9) | 1.8 (1.8, 1.9) | -0.0 (-0.1, 0.1) | 0.636 |
| Unadjusted | 18 | 1.9 (1.8, 2.0) | 1.9 (1.9, 2.0) | -0.0 (-0.1, 0.1) | 0.690 |
| Including post terms | 18 | 1.8 (1.7, 1.9) | 1.8 (1.8, 1.9) | -0.0 (-0.1, 0.1) | 0.798 |
| Main analysis (adjusted) | 25 | 2.0 (1.9, 2.2) | 2.1 (2.0, 2.1) | -0.0 (-0.1, 0.1) | 0.758 |
| Complete case | 25 | 2.1 (1.9, 2.2) | 2.1 (2.0, 2.2) | -0.0 (-0.2, 0.1) | 0.424 |
| Unadjusted | 25 | 2.1 (2.0, 2.2) | 2.1 (2.0, 2.1) | -0.0 (-0.1, 0.1) | 0.866 |
| Including post terms | 25 | 2.1 (1.9, 2.2) | 2.1 (2.0, 2.1) | -0.0 (-0.1, 0.1) | 0.820 |

**Table S22 Comparison of observed and predicted values for lean mass and fat mass by preterm/full term birth**

| | Total number of observations | Mean observed (SD), kg | Mean predicted (SD), kg | Mean difference (observed – predicted), kg |
|---|---|---|---|---|
| Lean mass | | | | |
| Preterm: | | | | |
| 9 years | 397 | 24.07 (2.98) | 24.01 (2.23) | 0.06 (3.54) |
| 9-13 years | 375 | 29.30 (4.20) | 29.66 (4.40) | -0.35 (3.49) |
| 13-15 years | 320 | 38.07 (6.10) | 37.48 (6.17) | 0.59 (2.88) |

| | | | | |
|---|---|---|---|---|
| 15-18 years | 430 | 44.84 (8.99) | 44.98 (7.07) | -0.14 (2.99) |
| 18-25 years | 259 | 46.27 (9.50) | 46.35 (6.93) | -0.08 (3.87) |
| Overall | 1781 | 35.93 (11.00) | 35.93 (10.34) | 0.00 (3.36) |
| Full term: 9 years | 6476 | 24.59 (3.24) | 24.49 (2.41) | 0.10 (3.70) |
| 9-13 years | 6251 | 29.77 (4.44) | 30.11 (4.69) | -0.33 (3.85) |
| 13-15 years | 5412 | 37.94 (6.48) | 37.50 (6.69) | 0.44 (2.91) |
| 15-18 years | 7999 | 44.03 (9.17) | 44.14 (7.20) | -0.11 (3.16) |
| 18-25 years | 4298 | 46.83 (9.92) | 46.85 (7.24) | -0.02 (3.93) |
| Overall | 30436 | 36.28 (10.93) | 36.28 (10.22) | 0.00 (3.51) |
| Log fat mass | | | | |
| Preterm: | | | | |
| 9 years | 397 | 1.89 (0.61) | 1.90 (0.58) | -0.01 (0.09) |
| 9-13 years | 375 | 2.24 (0.62) | 2.22 (0.58) | 0.02 (0.12) |
| 13-15 years | 320 | 2.36 (0.63) | 2.39 (0.59) | -0.03 (0.13) |
| 15-18 years | 430 | 2.48 (0.62) | 2.47 (0.60) | 0.01 (0.10) |
| 18-25 years | 259 | 2.96 (0.49) | 2.96 (0.45) | 0.00 (0.09) |
| Overall | 1781 | 2.34 (0.69) | 2.34 (0.65) | -0.00 (0.11) |
| Full term: 9 years | 6476 | 1.98 (0.58) | 1.99 (0.55) | -0.01 (0.09) |
| 9-13 years | 6251 | 2.31 (0.57) | 2.29 (0.54) | 0.02 (0.11) |
| 13-15 years | 5412 | 2.46 (0.59) | 2.48 (0.55) | -0.02 (0.13) |
| 15-18 years | 7999 | 2.63 (0.61) | 2.63 (0.58) | 0.01 (0.10) |
| 18-25 years | 4298 | 3.00 (0.48) | 3.00 (0.43) | 0.00 (0.09) |
| Overall | 30436 | 2.45 (0.66) | 2.45 (0.63) | 0.00 (0.11) |

**Table S23 Comparison of observed and predicted values for SBP, DBP and pulse rate by preterm/full term birth**

| | Total number of observations | Mean observed (SD), mmHg | Mean predicted (SD), mmHg | Mean difference (observed – predicted), mmHg/bpm |
|---|---|---|---|---|
| SBP | | | | |
| Preterm: | | | | |
| 7 years | 442 | 99.95 (9.65) | 100.15 (4.66) | -0.20 (7.59) |
| 7-12 years | 1130 | 105.65 (9.37) | 105.25 (5.45) | 0.40 (6.37) |
| 12-16 years | 949 | 114.09 (11.44) | 114.49 (7.21) | -0.40 (7.53) |
| 16-18 years | 194 | 119.95 (9.47) | 120.23 (5.87) | -0.28 (6.92) |
| 18-25 years | 256 | 117.71 (10.93) | 117.44 (6.81) | 0.27 (6.74) |
| Overall | 2971 | 109.47 (12.00) | 109.47 (8.84) | 0.00 (7.02) |
| Full term: 7 years | 7174 | 98.82 (9.15) | 98.96 (4.75) | -0.14 (7.08) |
| 7-12 years | 18670 | 103.99 (9.38) | 103.74 (5.56) | 0.25 (6.36) |
| 12-16 years | 16053 | 112.66 (11.90) | 112.89 (7.55) | -0.23 (7.69) |
| 16-18 years | 3738 | 117.00 (10.25) | 117.23 (6.69) | -0.23 (7.04) |
| 18-25 years | 4343 | 115.97 (11.28) | 115.79 (7.25) | 0.18 (6.66) |
| Overall | 49978 | 108.05 (12.08) | 108.05 (8.88) | -0.00 (6.99) |
| DBP | | | | |
| Preterm: | | | | |
| 7 years | 442 | 57.20 (6.82) | 57.89 (3.06) | -0.68 (6.87) |

| | | | | |
|---|---|---|---|---|
| 7-12 years | 1131 | 59.03 (7.21) | 57.83 (3.30) | 1.20 (5.68) |
| 12-16 years | 949 | 60.01 (9.10) | 61.10 (4.16) | -1.09 (6.56) |
| 16-18 years | 194 | 64.04 (6.08) | 64.83 (3.63) | -0.79 (6.27) |
| 18-25 years | 256 | 66.48 (7.95) | 65.93 (4.84) | 0.55 (6.90) |
| Overall | 2972 | 60.04 (8.19) | 60.04 (4.62) | -0.00 (6.38) |
| Full term: 7 years | 7172 | 56.44 (6.64) | 57.19 (2.94) | -0.75 (6.37) |
| 7-12 years | 18672 | 58.76 (7.05) | 57.51 (3.38) | 1.25 (5.50) |
| 12-16 years | 16053 | 59.99 (8.82) | 61.10 (4.30) | -1.11 (6.08) |
| 16-18 years | 3738 | 64.21 (6.39) | 64.65 (4.11) | -0.44 (7.19) |
| 18-25 years | 4343 | 66.55 (7.65) | 66.19 (4.46) | 0.36 (6.18) |
| Overall | 49978 | 59.90 (8.09) | 59.90 (4.82) | -0.00 (6.11) |
| Pulse rate | | | | |
| Preterm: | | | | |
| 7 years | 441 | 83.57 (11.32) | 83.11 (6.18) | 0.46 (7.67) |
| 7-12 years | 1147 | 74.96 (11.65) | 75.37 (6.99) | -0.41 (7.85) |
| 12-16 years | 949 | 72.64 (11.76) | 72.31 (7.23) | 0.33 (7.98) |
| 16-18 years | 194 | 65.70 (10.83) | 66.47 (6.93) | -0.76 (8.71) |
| 18-25 years | 256 | 67.26 (10.70) | 66.88 (6.03) | 0.39 (9.22) |
| Overall | 2987 | 74.23 (12.45) | 74.23 (8.31) | -0.00 (8.06) |
| Full term: 7 years | 7171 | 83.30 (10.74) | 82.82 (5.83) | 0.48 (7.57) |
| 7-12 years | 18911 | 75.66 (11.36) | 75.98 (6.60) | -0.31 (7.99) |
| 12-16 years | 16052 | 73.45 (11.40) | 73.28 (6.89) | 0.17 (7.66) |
| 16-18 years | 3738 | 66.07 (10.57) | 66.61 (6.64) | -0.54 (8.46) |
| 18-25 years | 4343 | 66.89 (10.16) | 66.46 (5.76) | 0.43 (8.56) |
| Overall | 50215 | 74.57 (12.09) | 74.57 (8.00) | 0.00 (7.92) |

**Table S24 Comparison of observed and predicted values for lipids by preterm/full term birth**

| | Total number of observations | Mean observed (SD), mmol/l | Mean predicted (SD), mmol/ | Mean difference (observed – predicted), mmol/l |
|---|---|---|---|---|
| HDL-c | | | | |
| Preterm: | | | | |
| Birth | 223 | 0.60 (0.29) | 0.60 (0.13) | -0.00 (0.15) |
| 7-18 years | 865 | 1.41 (0.33) | 1.41 (0.25) | -0.00 (0.13) |
| 18-25 years | 210 | 1.47 (0.39) | 1.47 (0.33) | 0.00 (0.11) |
| Overall | 1298 | 1.28 (0.45) | 1.28 (0.40) | 0.00 (0.13) |
| Full term Birth | 4401 | 0.53 (0.24) | 0.53 (0.11) | 0.00 (0.13) |
| 7-18 years | 14799 | 1.39 (0.32) | 1.39 (0.24) | -0.00 (0.13) |
| 18-25 years | 3475 | 1.50 (0.42) | 1.50 (0.35) | 0.00 (0.11) |
| Overall | 22675 | 1.24 (0.48) | 1.24 (0.43) | 0.00 (0.13) |
| Non-HDL-c | | | | |
| Preterm: | | | | |
| Birth | 223 | 1.33 (0.66) | 1.35 (0.52) | -0.02 (0.15) |
| 0-9 years | 296 | 2.84 (0.66) | 2.74 (0.43) | 0.10 (0.32) |
| 9-18 years | 569 | 2.62 (0.66) | 2.67 (0.54) | -0.05 (0.28) |
| 18-25 years | 210 | 2.85 (0.81) | 2.83 (0.67) | 0.02 (0.24) |

| | | | | |
|---|---|---|---|---|
| Overall | 1298 | 2.49 (0.87) | 2.49 (0.75) | -0.00 (0.27) |
| Full term Birth | 4400 | 1.30 (0.86) | 1.32 (0.67) | -0.02 (0.19) |
| 0-9 years | 4811 | 2.88 (0.63) | 2.78 (0.43) | 0.10 (0.30) |
| 9-18 years | 9989 | 2.66 (0.67) | 2.70 (0.53) | -0.04 (0.30) |
| 18-25 years | 3475 | 2.82 (0.84) | 2.81 (0.68) | 0.01 (0.25) |
| Overall | 22675 | 2.47 (0.93) | 2.47 (0.80) | -0.00 (0.28) |
| Log triglycerides | | | | |
| Preterm: | 221 | -0.89 (0.41) | -0.88 (0.18) | -0.01 (0.23) |
| Birth | | | | |
| 0-9 years | 296 | -0.06 (0.46) | -0.09 (0.20) | 0.03 (0.30) |
| 9-18 years | 569 | -0.15 (0.42) | -0.13 (0.24) | -0.02 (0.27) |
| 18-25 years | 210 | -0.17 (0.42) | -0.18 (0.20) | 0.02 (0.30) |
| Overall | 1296 | -0.26 (0.51) | -0.26 (0.36) | 0.00 (0.27) |
| Full term Birth | 4477 | -0.67 (0.45) | -0.67 (0.20) | -0.00 (0.25) |
| 0-9 years | 4811 | -0.04 (0.44) | -0.05 (0.20) | 0.01 (0.30) |
| 9-18 years | 9989 | -0.12 (0.43) | -0.12 (0.24) | -0.01 (0.28) |
| 18-25 years | 3474 | -0.14 (0.42) | -0.15 (0.21) | 0.01 (0.28) |
| Overall | 22751 | -0.22 (0.49) | -0.22 (0.32) | 0.00 (0.28) |

**Table S25 Comparison of observed and predicted values for glucose and insulin by preterm/full term birth**

| | Total number of observations | Mean observed (SD), mmol/l | Mean predicted (SD), mmol/l | Mean difference (observed – predicted), mmol/l |
|---|---|---|---|---|
| Glucose | | | | |
| Preterm: | | | | |
| 7-15 years | 327 | 4.27 (0.56) | 4.26 (0.18) | 0.01 (0.41) |
| 15-18 years | 294 | 5.15 (0.43) | 5.15 (0.22) | 0.00 (0.51) |
| 18-25 years | 210 | 5.27 (0.48) | 5.29 (0.20) | -0.02 (0.42) |
| Overall | 831 | 4.84 (0.67) | 4.84 (0.51) | -0.00 (0.45) |
| Full term: 7-15 years | 5706 | 4.30 (0.56) | 4.29 (0.20) | 0.01 (0.41) |
| 15-18 years | 5421 | 5.13 (0.42) | 5.15 (0.23) | -0.02 (0.49) |
| 18-25 years | 3466 | 5.24 (0.51) | 5.23 (0.23) | 0.01 (0.43) |
| Overall | 14593 | 4.83 (0.66) | 4.83 (0.49) | 0.00 (0.45) |
| Log insulin | | | | |
| Preterm: | | | | |
| Birth | 26 | 1.04 (0.48) | 1.04 (0.15) | -0.00 (0.34) |
| 9-15 years | 35 | 1.47 (0.61) | 1.47 (0.33) | 0.00 (0.29) |
| 15-18 years | 292 | 2.05 (0.59) | 2.05 (0.24) | 0.00 (0.46) |
| 18-25 years | 209 | 2.02 (0.62) | 2.03 (0.30) | -0.00 (0.38) |
| Overall | 562 | 1.96 (0.64) | 1.96 (0.36) | 0.00 (0.41) |
| Full term Birth | 613 | 1.10 (0.56) | 1.10 (0.18) | 0.00 (0.38) |
| 9-15 years | 874 | 1.66 (0.68) | 1.66 (0.38) | -0.00 (0.32) |
| 15-18 years | 5379 | 2.09 (0.54) | 2.10 (0.23) | -0.00 (0.43) |
| 18-25 years | 3467 | 2.03 (0.62) | 2.03 (0.30) | 0.00 (0.36) |
| Overall | 10333 | 1.98 (0.63) | 1.98 (0.37) | -0.00 (0.39) |

**Table S26 Summary of the number of participants included across each analysis**

| | | Pre-term | Full term |
|---|---|---|---|
| BMI | main analysis | 676 | 10534 |
| | complete case | 430 | 7051 |
| | including 41 weeks | 676 | 11413 |
| | excluding pregnancy complications | 456 | 10534 |
| | offspring males | 378 | 5309 |
| | offspring females | 298 | 5225 |
| Fat mass/lean mass | main analysis | 494 | 7386 |
| | complete case | 330 | 5180 |
| | including 41 weeks | 494 | 7992 |
| | excluding pregnancy complications | 329 | 7386 |
| | offspring males | 266 | 3584 |
| | offspring females | 228 | 3802 |
| SBP/DBP/pulse rate | main analysis | 559 | 8195 |
| | complete case | 368 | 5674 |
| | including 41 weeks | 559 | 8882 |
| | excluding pregnancy complications | 373 | 8195 |
| | offspring males | 310 | 4047 |
| | offspring females | 249 | 4148 |
| Lipids | main analysis | 542 | 8566 |
| | complete case | 345 | 5733 |
| | including 41 weeks | 542 | 9262 |
| | excluding pregnancy complications | 367 | 8566 |
| | offspring males | 303 | 4279 |
| | offspring females | 239 | 4287 |
| Glucose | main analysis | 417 | 6339 |
| | complete case | 280 | 4452 |
| | including 41 weeks | 417 | 6837 |
| | excluding pregnancy complications | 280 | 6339 |
| | offspring males | 233 | 3110 |
| | offspring females | 184 | 3229 |
| Insulin | main analysis | 311 | 4973 |
| | complete case | 214 | 3546 |
| | including 41 weeks | 311 | 5365 |
| | excluding pregnancy complications | 205 | 4973 |
| | offspring males | 161 | 2318 |
| | offspring females | 150 | 2655 |

**Table S27 Estimated mean difference in cardiometabolic risk factors per 1 week decrease in gestational age**

| Risk factors | Ages | MD (95% CI) per 1 week decrease in gestational age |
|---|---|---|
| BMI | Age 1yr (log BMI, kg/m2) | -0.007 (-0.023, 0.010) |
| | Age 3yr (log BMI, kg/m2) | -0.003 (-0.010, 0.003) |
| | Age 7yr (log BMI, kg/m2) | -0.002 (-0.004, 0.000) |
| | Age 9yr (log BMI, kg/m2) | -0.002 (-0.003, 0.000) |
| | Age 11yr (log BMI, kg/m2) | -0.002 (-0.003, 0.000) |
| | Age 13yr (log BMI, kg/m2) | -0.001 (-0.003, 0.000) |
| | Age 15yr (log BMI, kg/m2) | -0.001 (-0.003, 0.000) |
| | Age 18yr (log BMI, kg/m2) | -0.001 (-0.003, 0.000) |
| | Age 25yr (log BMI, kg/m2) | -0.001 (-0.004, 0.001) |
| Lean mass | Age 9yr (kg) | -0.105 (-0.153, -0.057) |
| | Age 11yr (kg) | -0.099 (-0.145, -0.053) |
| | Age 13yr (kg) | -0.094 (-0.171, -0.017) |
| | Age 15yr (kg) | -0.088 (-0.165, -0.011) |
| | Age 18yr (kg) | -0.139 (-0.219, -0.059) |
| | Age 25yr (kg) | -0.155 (-0.239, -0.071) |
| Fat mass (logged) | Age 9yr (kg) | -0.010 (-0.019, -0.002) |
| | Age 11yr (kg) | -0.008 (-0.014, -0.001) |
| | Age 13yr (kg) | -0.005 (-0.012, 0.002) |
| | Age 15yr (kg) | -0.007 (-0.014, 0.000) |
| | Age 18yr (kg) | -0.006 (-0.014, 0.001) |
| | Age 25yr (kg) | -0.003 (-0.011, 0.004) |
| SBP | Age 7yr (mmHg) | 0.188 (0.068, 0.307) |
| | Age 9yr (mmHg) | 0.205 (0.115, 0.295) |
| | Age 11yr (mmHg) | 0.223 (0.130, 0.317) |
| | Age 13yr (mmHg) | 0.212 (0.117, 0.308) |
| | Age 15yr (mmHg) | 0.172 (0.039, 0.306) |
| | Age 18yr (mmHg) | 0.363 (0.208, 0.519) |
| | Age 25yr (mmHg) | 0.116 (-0.069, 0.302) |
| DBP | Age 7yr (mmHg) | 0.115 (0.027, 0.202) |
| | Age 9yr (mmHg) | 0.076 (0.012, 0.139) |
| | Age 11yr (mmHg) | 0.036 (-0.032, 0.105) |
| | Age 13yr (mmHg) | -0.009 (-0.077, 0.059) |
| | Age 15yr (mmHg) | -0.060 (-0.166, 0.046) |
| | Age 18yr (mmHg) | 0.058 (-0.053, 0.169) |
| | Age 25yr (mmHg) | -0.079 (-0.232, 0.074) |
| Pulse rate | Age 7yr (bpm) | 0.118 (-0.024, 0.259) |
| | Age 9yr (bpm) | 0.068 (-0.038, 0.173) |
| | Age 11yr (bpm) | 0.018 (-0.089, 0.125) |
| | Age 13yr (bpm) | -0.054 (-0.163, 0.055) |
| | Age 15yr (bpm) | -0.146 (-0.296, 0.004) |
| | Age 18yr (bpm) | 0.212 (0.037, 0.386) |
| | Age 25yr (bpm) | -0.021 (-0.209, 0.167) |
| Triglycerides (logged) | Birth (mmol/l) | -0.051 (-0.059, -0.043) |

| | | |
|---|---|---|
| | Age 7yr (mmol/l) | -0.011 (-0.016, -0.006) |
| | Age 9yr (mmol/l) | 0.001 (-0.005, 0.007) |
| | Age 15yr (mmol/l) | -0.003 (-0.008, 0.002) |
| | Age 18yr (mmol/l) | -0.004 (-0.011, 0.002) |
| | Age 25yr (mmol/l) | -0.013 (-0.022, -0.004) |
| HDL-c | Birth (mmol/l) | 0.015 (0.010, 0.019) |
| | Age 7yr (mmol/l) | 0.003 (-0.002, 0.007) |
| | Age 9yr (mmol/l) | 0.002 (-0.002, 0.006) |
| | Age 15yr (mmol/l) | 0.000 (-0.004, 0.004) |
| | Age 18yr (mmol/l) | -0.001 (-0.005, 0.004) |
| | Age 25yr (mmol/l) | 0.002 (-0.006, 0.010) |
| Non-HDL-c | Birth (mmol/l) | -0.004 (-0.019, 0.011) |
| | Age 7yr (mmol/l) | 0.002 (-0.006, 0.010) |
| | Age 9yr (mmol/l) | 0.004 (-0.006, 0.014) |
| | Age 15yr (mmol/l) | -0.002 (-0.011, 0.007) |
| | Age 18yr (mmol/l) | -0.005 (-0.016, 0.006) |
| | Age 25yr (mmol/l) | 0.001 (-0.016, 0.017) |
| Glucose | Age 7yr (mmol/l) | 0.003 (-0.006, 0.012) |
| | Age 9yr (mmol/l) | 0.001 (-0.006, 0.008) |
| | Age 15yr (mmol/l) | -0.004 (-0.013, 0.005) |
| | Age 18yr (mmol/l) | 0.003 (-0.008, 0.014) |
| | Age 25yr (mmol/l) | 0.008 (-0.006, 0.021) |
| Insulin (logged) | Birth (mmol/l) | 0.035 (0.005, 0.065) |
| | Age 9yr (mmol/l) | -0.011 (-0.048, 0.025) |
| | Age 15yr (mmol/l) | -0.005 (-0.016, 0.006) |
| | Age 18yr (mmol/l) | -0.008 (-0.021, 0.004) |
| | Age 25yr (mmol/l) | -0.006 (-0.020, 0.008) |

Gestational age restricted to less than 42 weeks. All models adjusted for (and entered into the models as main effects and interactions with offspring age) offspring sex and maternal characteristics: age, parity, smoking status, alcohol intake, pre pregnancy BMI, HDP or preexisting hypertension, existing diabetes or GDM or glycosuria, ethnicity (white European compared to non-white European) and any treatments to help conception in this pregnancy (including IVF) (compared to none) .

**Figure S1. Estimated mean difference (95% CI) between those born preterm versus full term (blue, main analysis) and additionally adjusted for birthweight (red, sensitivity analysis)** Full terms restricted to less than 42 weeks. All models adjusted for (and entered into the models as main effects and interactions with offspring age) offspring sex and maternal characteristics: age, parity, smoking status, alcohol intake, pre pregnancy BMI, HDP or preexisting hypertension, existing diabetes or GDM or glycosuria, ethnicity (white European compared to non-white European) and any treatments to help conception in this pregnancy (including IVF) (compared to none). Estimates <0 mean lower values for preterm vs term, MD=mean difference**.**

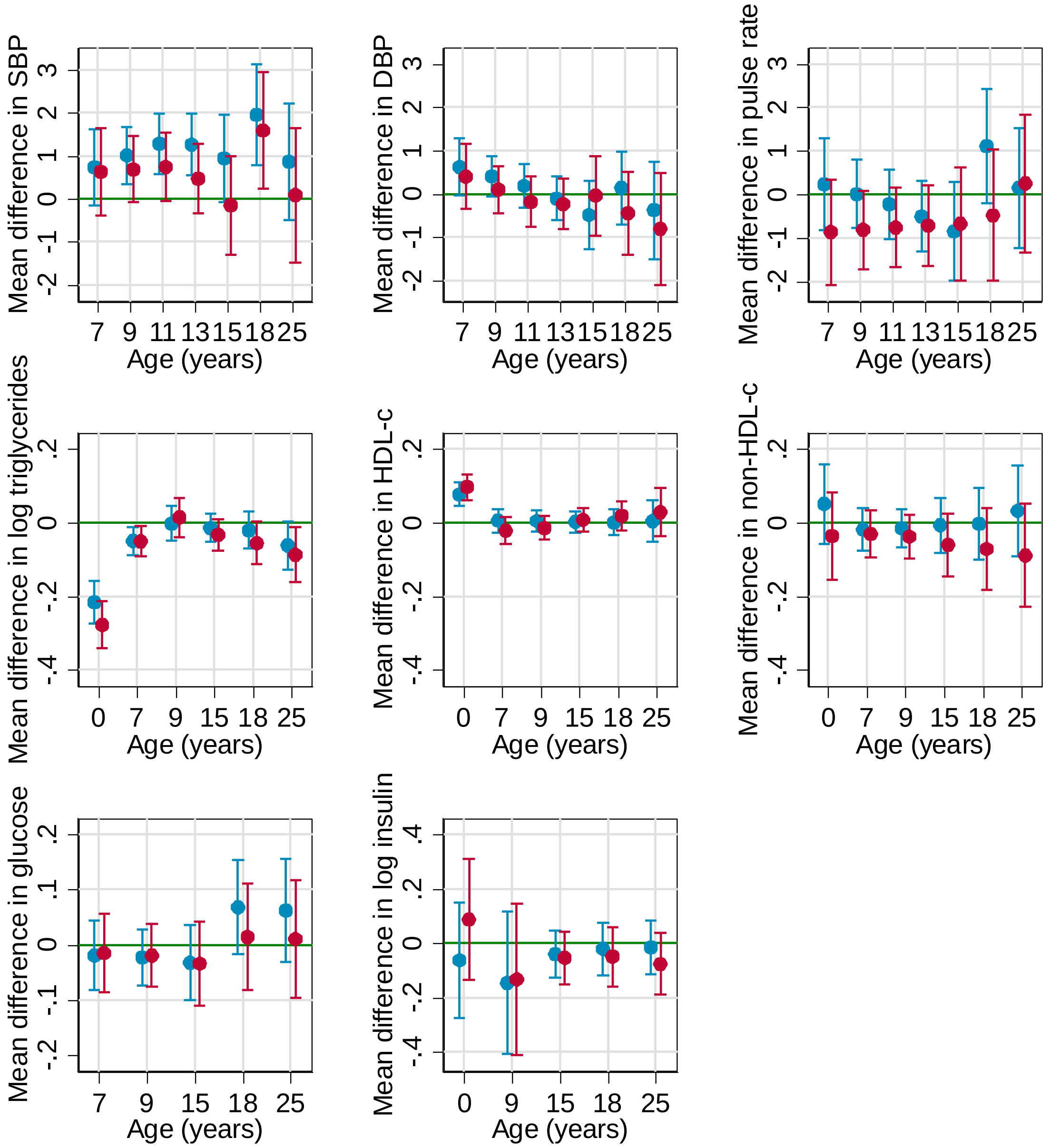

**Figure S2. Estimated mean difference (95% CI) between those born preterm versus full term in females (blue) and males (red)** Full terms restricted to less than 42 weeks. All models adjusted for (and entered into the models as main effects and interactions with offspring age) maternal characteristics: age, parity, smoking status, alcohol intake, pre pregnancy BMI, HDP or existing hypertension, existing diabetes or GDM or glycosuria, ethnicity (white European compared to non-white European) and any treatments to help conception in this pregnancy (including IVF) (compared to none). Preterm/full terms Ns for BMI, lean/fat mass, SBP/DBP/pulse rate. triglycerides/HDL/HDL, glucose and insulin were females: 372/5282, 261/3557, 305/4023. 299/4254, 229/3091, 160/2301; males 294/5209, 225/3790, 245/4136. 235/4275, 182/3220, 148/2647.

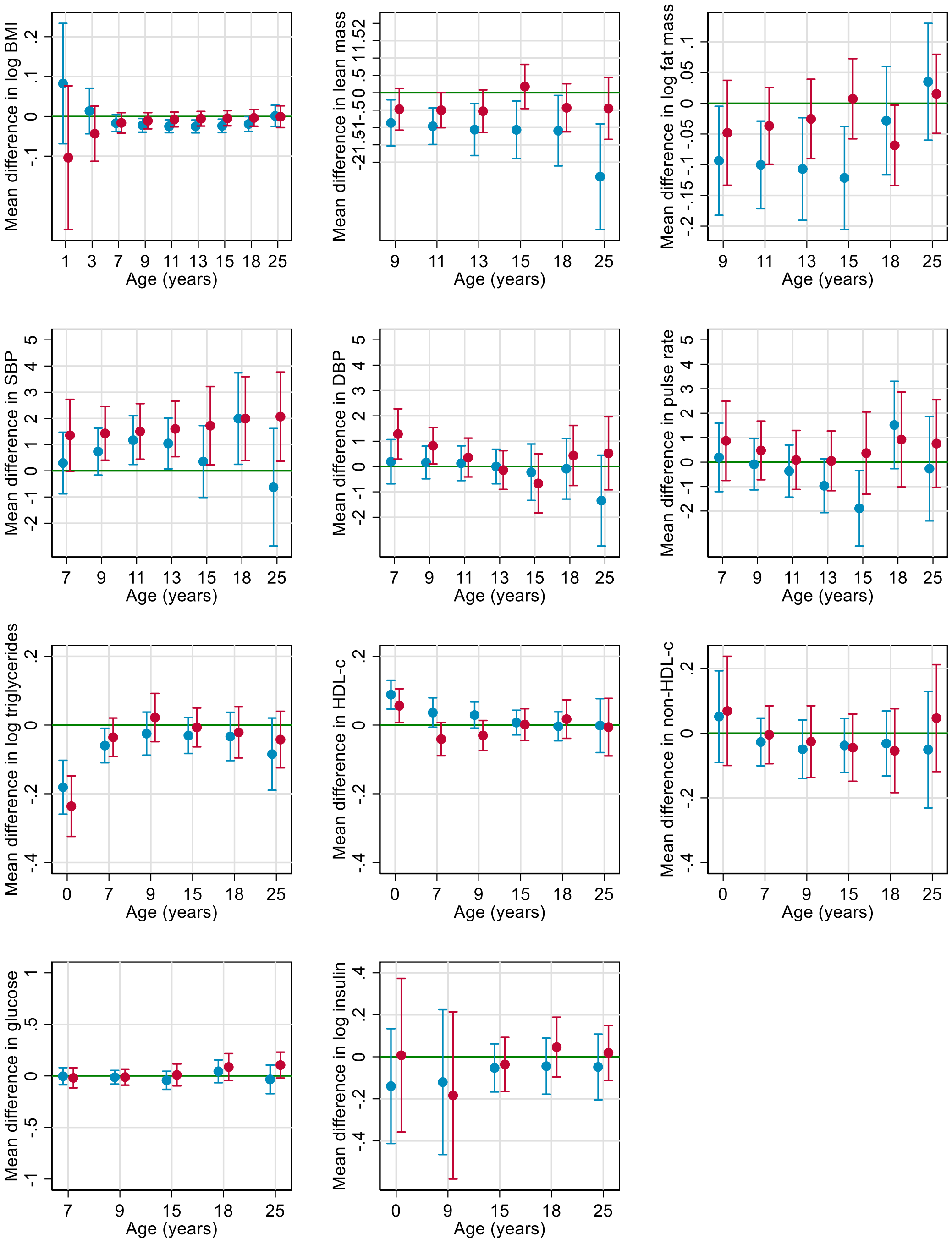

**Figure S3. Estimated mean difference (95% CI) between those born preterm versus full term (blue, main analysis) and when only including preterm births who had a spontaneous labour (red, spontaneous labour)** Full terms restricted to less than 42 weeks. Pre terms births were restricted to when the method of delivery was ‘spontaneous’. Preterm/full terms Ns for BMI, lean/fat mass, SBP/DBP/pulse rate. triglycerides/HDL/HDL, glucose and insulin in the main analysis: 676/10534, 493/7380, 559/8195. 542/8566, 417/6339, 311/4973; spontaneous subgroup: 382/10534, 279/7380, 319/8195. 319/8566, 239/6338, 180/4973. All models adjusted for (and entered into the models as main effects and interactions with offspring age) offspring sex and maternal characteristics: age, parity, smoking status, alcohol intake, pre pregnancy BMI, ethnicity (white European compared to non-white European) and any treatments to help conception in this pregnancy (including IVF) (compared to none)

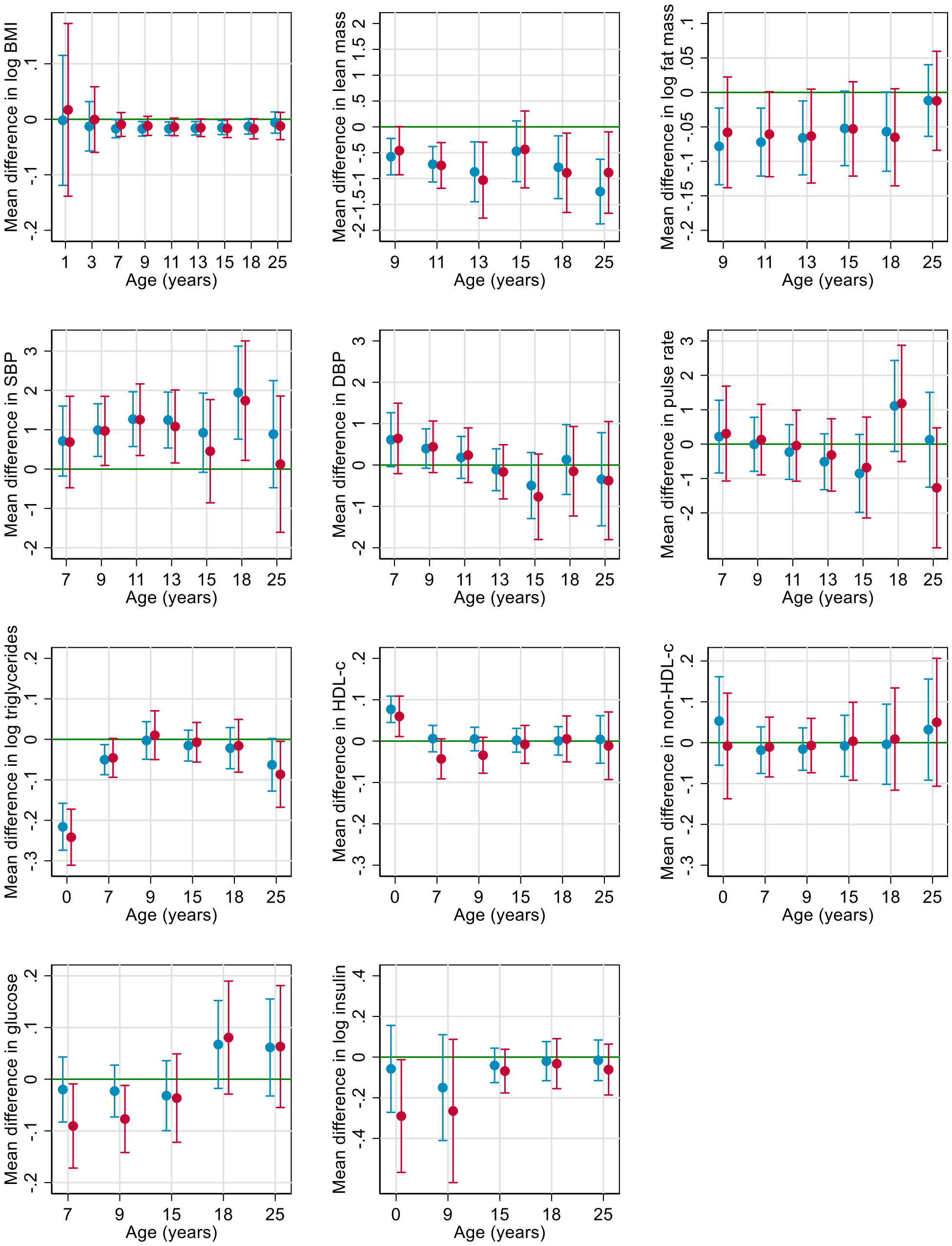

Supplement: Supplementary file 1 — Tables S1–S27 Figures S1–S3 [file JAH3-14-e030823-s001.pdf]
